# Polyphenols from *Dichrostachys cinerea* Fruits Anti-Inflammatory, Analgesic, and Antioxidant Capacity in Freund’s Adjuvant-Induced Arthritic Rat Model

**DOI:** 10.3390/molecules27175445

**Published:** 2022-08-25

**Authors:** Gisèle Atsang à Kiki, Raluca Maria Pop, Octavia Sabin, Ioana Corina Bocsan, Veronica Sanda Chedea, Sonia Ancuța Socaci, Alina Elena Pârvu, Egre Finsia, Takvou Francis, Zramah Mathieu, Anca Dana Buzoianu

**Affiliations:** 1 Department of Biological Sciences, Faculty of Science, University of Maroua, Maroua P.O. Box 814, Cameroon; 2 Department of Pharmacology, Toxicology and Clinical Pharmacology, Faculty of Medicine, “Iuliu Hatieganu” University of Medicine and Pharmacy, 400012 Cluj-Napoca, Romania; 3 Research Station for Viticulture and Enology Blaj (SCDVV Blaj), 515400 Blaj, Romania; 4 Department of Food Science, University of Agricultural Sciences and Veterinary Medicine of Cluj-Napoca, 400372 Cluj-Napoca, Romania; 5 Department of Pathophysiology, Faculty of Medicine, “Iuliu Hatieganu” University of Medicine and Pharmacy, 400012 Cluj-Napoca, Romania

**Keywords:** *Dichrostachys cinerea*, phenolic compounds, analgesic, anti-inflammatory, antioxidant

## Abstract

*Dichrostachys cinerea* (L.) Wigth & Arn. (DC) is widely used in traditional medicine against several inflammatory diseases, especially rheumatoid arthritis, because of its antioxidant and anti-inflammatory effects. This study aimed to characterize the polyphenol-rich DC fruit extracts and investigate the analgesic, anti-inflammatory, and antioxidant effects in a rat inflammation model induced by complete Freund’s adjuvant (CFA). Water and ethanolic extracts were characterized using liquid chromatography coupled with mass spectrometry (LC-MS), Fourier-transform infrared (FTIR) spectroscopy, and gas chromatography coupled with mass spectrometry (GC-MS). The polyphenol-rich extracts were administered in three different concentrations for 30 days. Pain threshold, thermal hyperalgesia, edema, and serum biomarkers specific to inflammatory processes or oxidative stress were evaluated. Both extracts were rich in polyphenolic compounds, mainly flavan-3-ols, proanthocyanidins, and flavone glycosides, which had important in vitro antioxidant capacity. DC fruit extracts administration had the maximum antinociceptive and anti-inflammatory effects after one day since the CFA injection and showed promising results for long-term use as well. The measurement of pro-inflammatory cytokines, cortisol, and oxidative stress parameters showed that DC extracts significantly reduced these parameters, being dose and extract-type dependent. These results showed potential anti-inflammatory, analgesic, and antioxidative properties and revealed the necessity of using a standardized polyphenolic DC extract to avoid result variability.

## 1. Introduction

Rheumatoid arthritis (RA) affects approximately 1% of people worldwide, and little data is available about the disease burden in Africa. Despite potentially fatal systemic manifestations, RA is frequently regarded as a minor health issue, and more effort is needed for its management and research in Africa [1]. The major pathophysiological component of RA is the chronic inflammatory process, which includes multiple systemic changes such as synovial hyperplasia because of increased proliferative cellular infiltrates of leukocytes, an abnormal increase in several pro-inflammatory cytokines (particularly Interleukin 6 (IL-6), Interleukin-1 beta (IL-1β), and Tumor Necrosis Factor Alpha (TNF-α)), consequent cartilage and bone destruction with swelling, deformation, and loss of function of joints [2,3]. The current therapy for RA is based generally on non-steroidal or steroidal anti-inflammatory drugs and immunosuppressive drugs. Their use is, however, limited mostly because of their adverse reactions such as gastrointestinal ulcers, hemorrhage, myelosuppression, hypertension exacerbation, neutropenia, and others [4]. That is why numerous studies are focusing on developing effective, safe, or alternative drugs capable of preventing and reducing these side effects.

Dietary polyphenols are considered important natural antioxidants readily available in human diets such as vegetables, fruits, grains, tea, or their derived products that can have a major impact on health. Literature studies bring important evidence of these dietary phenolic compounds’ roles in lowering risk factors or in preventing the onset of various degenerative diseases such as diabetes, neurodegenerative diseases, cardiovascular diseases, metabolic disorders, obesity, cancers, and others [5,6,7].

The culture of using medicinal plants and natural products for treating or preventing different diseases has ancient roots and is continuing these days too in many parts of the world. One of these plants is *Dichrostachys cinerea* (L.) Wigth & Arn. (DC) (species also named *Cailliea dichrostachys* or *Dichrostachys glomerata*)*,* a terrestrial herbaceous plant of the family Mimosaceae. It is widespread in Africa and India, and is considered an invasive species in the Caribbean. *Dichrostachys cinerea*’s bark, roots, and fruit pods are used in traditional medicine as remedies against common health problems such as fever, pain, and diarrhea, but it also has laxative properties [8]. It is also used in some populations to prevent sexually transmitted diseases [9,10], for cicatrizing effects [11,12], or as an anti-inflammatory agent [13]. Currently, most of the studies have predominantly investigated the pharmacological activities of roots, bark, and leaves. To our knowledge, there are few works regarding fruit composition and their pharmacological activities [9,12,14,15,16,17]. Additionally, the chemical composition is reported in terms of total polyphenolic compounds, total flavonoids, total alkaloids, carbohydrates, total saponins, steroids, proteins, and amino acids [15,18], and not on the exact chemical profile. Even though there is evidence of the potential antioxidant and anti-inflammatory effects of DC [12,14,19], these data are limited. More studies are necessary to understand DC’s polyphenol mechanism of action in reducing the inflammatory processes.

Accordingly, this study aimed to characterize DC water and ethanolic fruit extracts and to reveal potential components with antioxidant and anti-inflammatory effects. Moreover, the antiarthritic effects of DG were investigated by using the induced complete Freund’s adjuvant (CFA) model in rats, knowing that induced arthritis by CFA in rats is one of the election models to study the anti-inflammatory effects of drugs and plant extracts. Since pain and stress are important components of arthritis, the effect on cortisol, a biomarker of acute stress, and the analgesic effect of DG extracts were also investigated.

## 2. Results

### 2.1. Total Polyphenol Content and In Vitro Antioxidant Capacity

Total polyphenols content (TPC) and relative 2,2-diphenyl1-picrylhydrazyl (DPPH) radical scavenging capacity of Dichrostachys cinerea fruit water and ethanolic extracts are presented in Table 1.

### 2.2. Liquid Chromatography–Diode Array Detection–Electro-Spray Ionization Mass Spectrometry (HPLC-DAD-ESI MS) Analysis

In total, 17 compounds were tentatively identified as presented in Table 2. These compounds belonged to different phenolic classes, such as the following: flavonols (two compounds), flavon-3-ols (four), flavan -3-ol glycoside (one), flavone glycosides (three), isoflavones (one), proanthocyanidins (five) and stilbenoids (one). The compound identification was performed according to their retention time, UV-VIS spectra, and their protonated molecules [M+H]+ (Supplementary Materials and literature data).

### 2.3. Fourier Transform Infrared Spectroscopy (FTIR) Analysis

**Figure 1:**
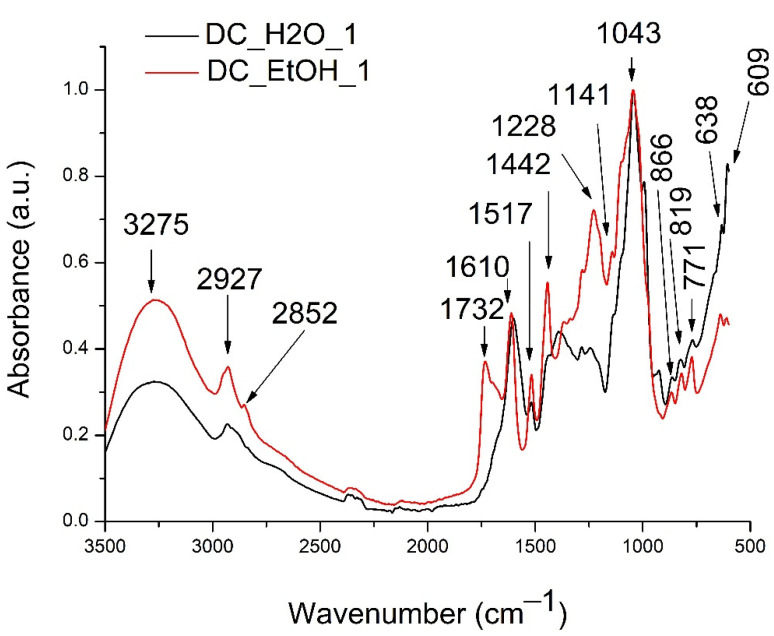
*Dichrostachys cinerea* water and ethanolic general FTIR spectra (3500–500 cm^−1^).

FTIR analysis was performed to identify other compound classes that were extracted from de DC in the water and the ethanolic extracts. The presence of other compounds is based on the specific absorption spectrum of different functional groups and chemical bonds existing in the mixture of compounds extracted. The representative general FTIR spectra recorded between 3500–500 cm^−1^ of DC_H2O_1 and DC_EtOH_1 are presented in Figure 1.

*Dichrostachys cinerea* water and ethanolic extracts had similar spectra, having 15 major peaks, among which some presented higher absorption intensities in the ethanolic extract. The intense absorption peak at 3275 cm^−1^, which was one of the peaks with higher intensity in the ethanolic extract, could be attributed to the hydroxyl group specific for phenolics and phenolic glycosides [30,31]. The next bands at 2927 cm^−1^ and 2852 can be assigned to the alkane –CH bond [30,31]. The medium intensity band identified only in the methanolic extract at 1732 cm^−1^ and 1610 cm^−1^ could be attributed to could be assigned to the C=O stretching vibration in the lipid esters [32]. They could also be assigned to C=O and C=C carbonyl groups stretching vibrations from phenolic compounds [33,34]. Further, the absorption bands characteristic of the amino group or the strong skeletal bands specific to aromatic compounds in 1600–1300 were confirmed by peaks between 1650–1350 cm^−1^ [30,31]. The high-intensity band at 1043 cm^−1^ found between 1170–930 cm^−1^ can be assigned to flavonoids and possibly polysaccharides [35]. Next, minor peaks between 870–810 cm^−1^ could be assigned to polysaccharides’ skeletal stretching vibrations [31,36], while the absorption peaks found between 550–750 cm^−1^ can be assigned to protein absorption [31].

### 2.4. In-Tube Extraction Dynamic Headspace Gas-Chromatography–Mass Spectrometry (ITEX/GC-MS) Analysis

The GC-MS analysis was performed to determine the extract’s volatile compounds. The water extract (DC_H2O_1) had no volatile compounds, while the ethanolic extract (DC_EtOH_1) contained 4 compounds as observed in Table 3.

### 2.5. Anti-Inflammatory Effect of Dichrostachys Cinerea Fruit Extracts on Rats Paw Edema

**Figure 2:**
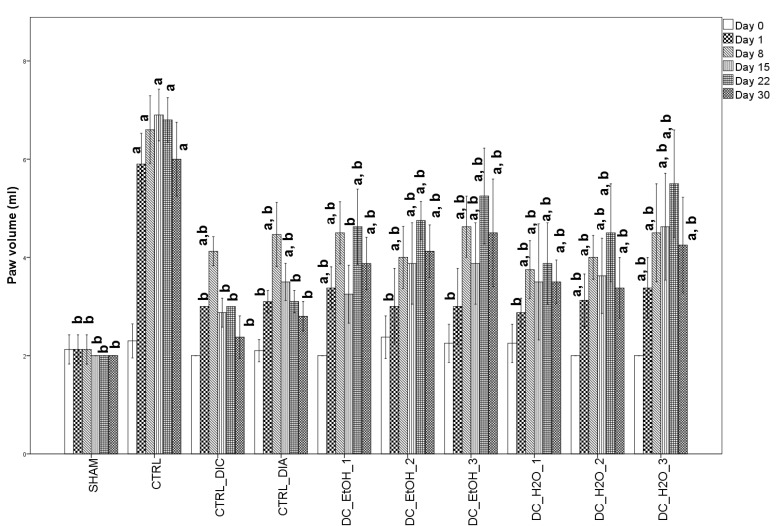
Effect of *Dichrostachys cinerea* water and ethanolic extracts on paw edema. Data are presented as means ± SD (n = 10 for each group). For comparison between groups, one-way analysis of variance (ANOVA) with Tukey multiple comparison tests were used. Superscript ‘a’ indicates a significant difference (*p* < 0.05) as compared to the SHAM group while superscript ‘b’ indicates a significant difference (*p* < 0.05) as compared to the CTRL group.

All rats with CFA-induced inflammation had marked peripheral paw edema, strongly visible one day after CFA injection, which continued to have a progressive increase until day 8 in the case of both positive control groups (Figure 2). The administration of DC ethanolic and water extracts showed the maximum edema later, on day 22, with a linear progression only for the DC_H2O_3 group. 

When compared with the CTRL group, 1 day after injection, all administrated substances helped prevent inflammation, having significantly lower values (*p* = 0.001). When compared to the SHAM, CTRL_DIC, DC_EtOH_2, DC_EtOH_3, and DC_H2O_1 groups were not statistically different (Figure 2). This indicates that after one day, the above DC fruit extracts prevented inflammation. Further, after 8 days from CFA paw injection, the inflammation continued to increase progressively, with all groups having significantly higher values when compared to the SHAM group and significantly lower when compared to the CTRL group, suggesting the extracts’ anti-inflammatory effect (Figure 2). When compared with diclofenac and diazepam groups, DC extracts had similar effects, the differences being statistically non-significant. At 15 days from CFA injection, DC_DIC, and DC_EtOH_1 had the strongest anti-inflammatory effects, having no significant differences when compared to the SHAM group. All other groups also had lower significant values when compared to CTRL (*p* = 0.001) (Figure 2). When compared with diclofenac, the ethanolic DC extract and the first two concentrations of the DC water extract had higher values but were not significantly different, suggesting similar effects (Figure 2). At 22 and 30 days since CFA injection, the anti-inflammatory effect was validated only for diclofenac and diazepam, groups that had higher values than the SHAM group, but with no statistical difference. At 22 days, only the administration of DC_H20_1 extract had a similar effect to diclofenac or diazepam administration (no statistically significant difference). At 30 days, DC_H2O_1 and DC_H2O_2 had similar effects with diclofenac and diazepam, while DC_EtOH_1 was only with diazepam (Figure 2).

### 2.6. Antinociceptive Effect of Dichrostachys Cinerea Fruit Extracts

**Figure 3:**
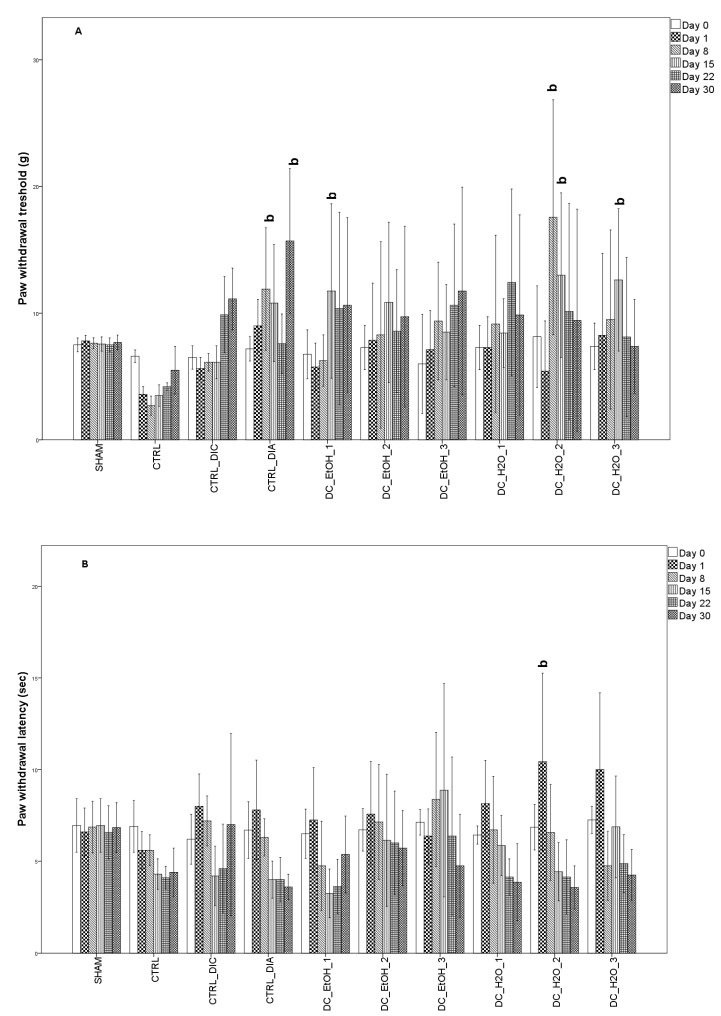
Effect of aqueous and ethanolic extract of *Dichrostachys cinerea* on mechano-allodynia (Analgesy-meter test) (**A**) and thermic induced pain (Hot plate test) (**B**). Data are presented as means ± SD (n = 10 for each group). For comparison between groups, one-way analysis of variance (ANOVA) with Tukey multiple comparison tests were used. Superscript ‘b’ indicates a significant difference (*p* < 0.05) as compared to the CTRL group.

As previously observed, the inflammatory model for rats was successfully achieved, with obvious signs of local inflammation (increased paw volume) being observed in the injected paws. As previously reported, inflammation induced by CFA is characterized by inflammatory pain as well. The next figure represents the antinociceptive effect of diclofenac, diazepam, and DC ethanolic and water administration against mechanical compression (Figure 3A) and thermal-induced pain (Figure 3B). Diclofenac was used in an ineffective dose (7.5 mg/kg) and had no significant effect in increasing the mechanical threshold or paw withdrawal threshold. Diazepam (0.5 mg/kg) had a significant effect only against mechanical compression. Administration of DC_H2O_2 extract had a significant analgesic effect against mechanical compression at 8 days after CFA administration, while at 15 days from CFA administration, DC_EtOH_1, DC_H2O_2, and DC_H2O_3 had a similar analgesic effect. Regarding DC administration against thermal-induced pain, it was observed that after 1 day after CFA injection, DC_H2O_2 administration significantly increased the pain threshold. Further, a similar effect was observed after the administration of DG_EtOH_3 after 15 days from the CFA injection.

### 2.7. Effect of Dichrostachys Cinerea Fruit Extracts on the Production of Pro-Inflammatory Cytokines

**Figure 4:**
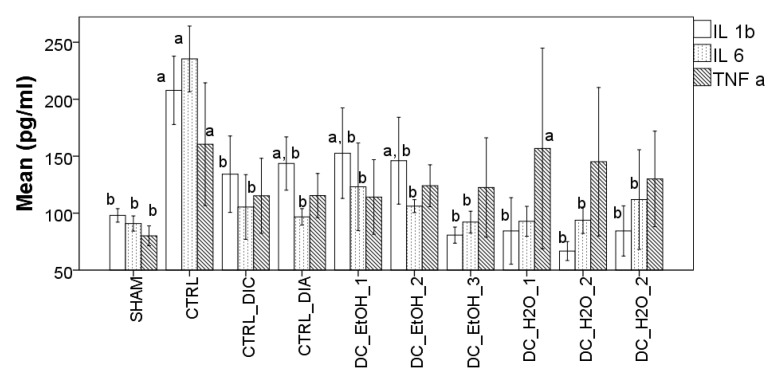
Effect of *Dichrostachys cinerea* fruit extracts on the inflammatory cytokines TNF-α, IL-1β, and IL-6 in CFA-induced arthritic rats. Serum inflammatory cytokines values are expressed as mean ± standard error of the mean (n = 10). Superscript ‘a’ indicates a significant difference (*p* < 0.05) as compared to the SHAM group while superscript ‘b’ indicates a significant difference (*p* < 0.05) as compared to the CTRL group as performed by one-way ANOVA, Tukey’s multiple comparisons test.

To further check the anti-inflammatory potential of DC in the CFA-induced arthritic rat model, the levels of pro-inflammatory serum cytokines TNF-α, IL-1β, and IL-6 were measured. As observed in Figure 4, the levels of serum TNF-α, IL-1β, and IL-6 identified in the CTRL group were significantly higher than those identified in the SHAM group. Administration of diclofenac, diazepam, and DC extracts in the CFA-induced inflammation groups significantly reduced the levels of IL-1β and IL-6. The same tendency was observed for TNF-α as well, but with no statistical significance (Figure 4).

### 2.8. Effect of Dichrostachys Cinerea Fruit Extracts on the Production of Cortisol

**Figure 5:**
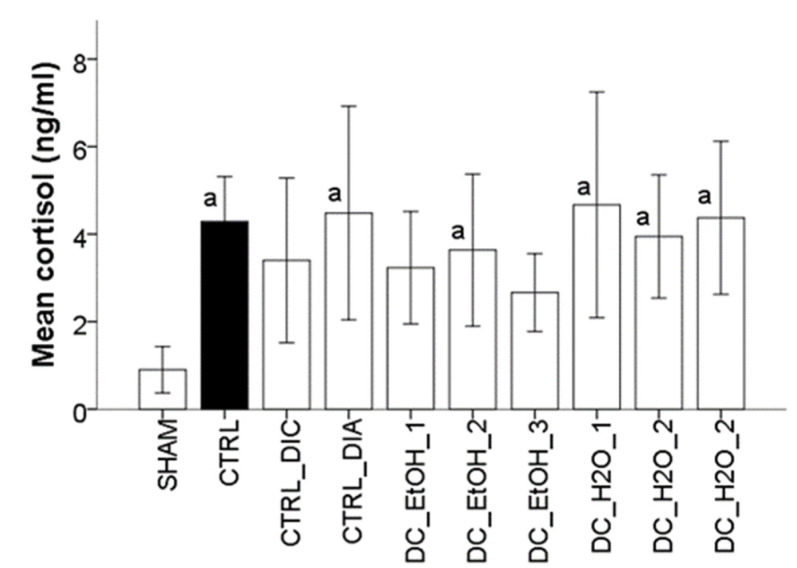
Effect of *Dichrostachys cinerea* fruit extracts on serum cortisol in CFA-induced arthritic rats. Serum inflammatory cytokines values are expressed as mean ± standard error of the mean (n = 10). Superscript ‘a’ indicates a significant difference (*p* < 0.05) as compared to the SHAM group as performed by one-way ANOVA, Tukey’s multiple comparisons test.

The stress as a consequence of CFA-induced inflammation in rats was investigated by measuring the levels of serum cortisol. When compared to the SHAM group, it was observed that cortisol levels were higher in all CFA groups (Figure 5). Among the administrated substances, diclofenac, first and third concentrations of DC ethanolic extracts had the best results in preventing stress. In these cases, the serum values of cortisol had no statistical significant change when compared with the SHAM group.

### 2.9. Effect of Dichrostachys Cinerea Fruit Extracts on Oxidative Stress Parameters

The effect of DC fruit extracts on the oxidative stress parameters was investigated by measuring malondialdehyde (MDA), nitric oxide (NO), and total thiols (THIOL), total antioxidant capacity (TAC), total oxidative status (TOS), and oxidative stability index (OSI). Administration of CFA led to a significant increase in all investigated parameters of oxidative stress, as observed in Table 4. Diclofenac sodium administration reduced the oxidative stress parameters as compared to the CTRL group, but with statistical significance only for NO. Administration of diazepam, DC_EtOH_1, DC_EtOH_2, and DC_H2O_2 had the same reduction tendency but with a statistically significant difference only for NO and MDA parameters, as compared with CTRL. The best results were registered for the lowest concentration of DC ethanolic extract DC_EtOH_3 and for the highest and lowest concentrations of DC water extracts (DC_H2O_1 and DC_H2O_3), which presented statistically significant differences for all parameters except total thiols. TAC was also measured but because the results were not statistically significant, having values between 1.08 and 1.09, these are not presented in Table 4. However, the values were used for the OSI calculation.

## 3. Discussion

The phytochemical analysis of DC fruits showed that the extraction solvent is influencing the compound’s concentration and composition. The ethanolic extract had approximately 1.4 times higher values of TPC when compared with the water extract. When compared with literature data, the results obtained in our study for the ethanolic extract were 1.3 higher than the one reported for DC fruits [37]. This difference can be explained by the fact that DC fruits were extracted using a mixture of 70/30 *v*/*v* of methanol/water [37], a mixture with a different polarity. *Dichrostachys cinerea* leaves methanolic extract were reported to contain approximately 1.9 times higher TPC than the ethanolic fruit extract, while roots extract approximately 1.4 times lower quantity [10]. Further, both extracts showed high antioxidant activities. It was observed that the higher phenolic content corresponded to the higher antioxidant capacity, as reported in the case of many other plant extracts [38]. Knowing that the antioxidant capacity is strongly correlated with the phenolic compounds profile, especially with the number of hydroxyl groups, their position, and the substitution type on the aromatic ring [39], the extracts’ strong antioxidant capacity was explained by the variety of phenolic compounds as determined by HPLC-MS (e.g., proanthocyanidins, flavonols, flavan-3-ol glycosides). The presence of polyphenols was also shown by the FTIR analysis. The presence of polysaccharides was also confirmed by the FTIR spectra.

Numerous in vitro studies already showed the high antioxidant potential of polyphenols plant extracts but also underlined the importance of their concentration in inducing both antioxidant and pro-oxidant effects, these activities being time and dose-dependent [40,41], so three different concentrations of both ethanolic and water s extracts of DC were tested using an experimental model of polyarthritis induced by CFA on rats, a model which is widely used for preclinical testing of numerous anti-arthritic agents [9].

In the present study, DC extracts treatment showed antiarthritic potential in all the inflammatory parameters. It significantly decreased the inflammation in treated animals by reducing the paw volume. It was observed that the best results were obtained after 24 h since CFA induction for groups treated with 102.84 mg GAE/g (DC_EtOH_2) and 51.42 mg GAE/g (DC_EtOH_3) of the ethanolic extract and for the group treated with 150.96 mg GAE/g (DC_H2O_1) of the water extract suggesting a better DC anti-inflammatory effect in acute inflammation. The best results on long-term treatment were obtained after administration of 150.96 mg GAE/g and 75.48 mg GAE/g of the water extract, highlighting the importance of bioactive compound concentrations to obtain the pharmacological effect. The anti-inflammatory and analgesic effect of DC was previously reported by Susithra and Jayakumari (2018) who investigated the DC leaf, stem bark, and root extracts using both acute and chronic inflammation models induced by carrageenan or cotton pellet granuloma, respectively [14]. Even if they did not investigate the fruit extracts, their results also indicated that leaf, stem bark, and root extracts had a better effect in the acute model and were ineffective in the chronic model [14]. The same anti-inflammatory effects were reported by Hassan et al. (2012) for the leaves of DC rich in saponins using a carrageenan inflammation rat model [13]. Further, the analgesic effect of DC extracts could be investigated since CFA injection induces specific modifications in transduction sensitivity with hyperalgesia and allodynia association. In our study, a significant analgesic effect was registered for DC_H2O_2 extract (102.84 mg GAE/g) at only one day since CFA administration indicating maximum efficiency in the acute thermal hyperalgesia induced by inflammation tested by thermic induced pain control, while in the mechanical hyperalgesia significant results were observed later, after 8 days from CFA injection. Our previous studies also indicated the analgesic effect of DC fruits water extract, but in a different model of pain (induced by acetic acid) and acute inflammation (induced by carrageenan) that the one presented in this study (induced by CFA) [12]. *Dychrostachis cinerea* analgesic effect was also reported in the literature for DC leaf, roots, or bark extracts in different acute inflammatory pain models [8,42], but to our knowledge, there are no references regarding the analgesic effect in chronic pain models.

Further, knowing that polyphenols possess the ability to reduce inflammation through their antioxidant properties, the ability to interfere with oxidative stress signaling, and the ability to suppress the pro-inflammatory signaling [7], the anti-inflammatory and antioxidant capacity of DC fruit extract rich in polyphenols was investigated by measuring IL-6, IL-1β and TNF-α, cortisol (inflammatory markers) and TOS, TAR, OSI, NO, MDA and total thiols (oxidative stress markers).

The increase in inflammatory cells has important significance in the pathogenesis of arthritis through pro-inflammatory cytokines production, which aggravates joint tissue pathology. Differentiation and activation of osteoclasts are amplified by TNF-α and IL-1 1 and 6. TNF-α plays an important and decisive role in endothelial cell adhesion molecules, angiogenesis, suppression of regulatory T-cells, protection of synovial fibroblasts, and induction of pain, while cartilage lesions and delayed healing lesions are due to IL-1β [43]. In the present study, it was observed that DC fruit extracts significantly reduced IL6 and IL1b production and exhibited the same tendency on TNF-a but with no statistical difference. Next, cortisol production is triggered by stress, and it has an essential anti-inflammatory effect. The results of this study revealed that ethanolic extracts of DC had a substantial effect on reducing cortisol levels. So far, to our knowledge, these are the first data concerning the effect of DC fruit extracts on these inflammatory biomarkers using a CFA-induced inflammation rat model. In addition to inflammatory processes, oxidative stress is also an important mechanism that contributes to the progression of destructive processes such as proliferative synovitis or articular degradation in arthritic rats [44]. Among oxidative stress parameters, increased lipid peroxidation levels are correlated to a reduced antioxidant capacity and thus increased oxidative tresss in these rats [44]. Accordingly, MDA can be used as a representative biomarker for lipid peroxidation status [44]. Nitric oxide, another oxidative stress biomarker, has its production regulated through the nitric oxide synthases (NOS), resulting in the production of neuronal, endothelial, and inducible isoforms [45]. The inducible isoform, highly expressed in macrophages, if activated can lead to organ destruction pathophysiological processes characteristic of inflammatory and autoimmune diseases. Therefore, the inhibition of the NOS inducible isoforms will result in decreased NOx production with a positive effect on acute inflammation [46]. Moreover, a low antioxidant level can be correlated to RA severity being considered an important risk factor in RA [44,47]. Accordingly, total oxidant status (TOS) is considered a pro-oxidant marker useful in the evaluation of the overall oxidation state. Opposite, total antioxidant status (TAS) is used to evaluate the overall antioxidant level [48]. Further, the oxidative stress index (OSI) is reflecting the imbalance between oxidation and antioxidant status since is obtained by calculating the ratio between TOS and TAC [48,49]. Finally, Thiols are useful in the defense system induced by oxidative stress via biochemical alterations, being the most vulnerable targets of ROS and related oxidants [48].

Within this context, the antioxidant effectiveness of DC extracts was assessed by measuring the serum values for MDA, TOS, OSI, NO, and TIOLS. From the results, it was observed that all extracts significantly reduced lipid peroxidation and NOx production. Total oxidative status and oxidative stress index were also reduced, with significant values for the lowest ethanolic DC extract (51.42 mg GAE/g sample) and the highest and lowest water DC extract (150, 96, and 37.74 mg GAE/g sample).

The main strength of the present study was the comparative analysis of the analgesic, antioxidant, and anti-inflammatory effects of three different concentrations of DC water and ethanolic extracts, as entire compounds, in CFA-induced experimental inflammation. The potential limitations of this study are referring firstly to a histological examination and secondly to the measurement of phenolic compounds metabolites to document their distribution in serum or tissue to validate DC polyphenol’s bioavailability and metabolism.

## 4. Materials and Methods

### 4.1. Chemicals

Complete Freund’s adjuvant (CFA) was purchased from Sigma-Aldrich (St. Louis, MO, USA). Sodium diclofenac (DIC) (Terapia S.A., Cluj-Napoca, Romania), diazepam (DIA) (Terapia S.A., Romania), and saline solution were purchased from the local pharmacy. Absolute ethanol was purchased from Merck (Darmstadt, Germany). All chemicals were of analytical grade.

### 4.2. Plant Material

Dried DC fruits were purchased from Yaounde market, Cameroon, Africa where these were commercialized as food supplements. Specialists from the 8594 SRF quality control laboratory of National Herbarium (part of the Institute of Agricultural Research for Development, Ministry of Scientific Research, Cameroon) performed the plant identification.

### 4.3. Fruits Ethanolic and Water Extraction

Fruits extracts were obtained by mixing 250 g of grounded DC with 1 L of distilled water or ethanol, to further obtain the aqueous and ethanolic extracts, respectively. The mixtures were further sonicated for 30 min at room temperature followed by filtration (Whatman filter paper no. 3). The pellets were resuspended again in 1 L of water and ethanol, respectively, and sonicated again for 30 min. The aqueous extract was concentrated to a final volume of 800 mL using a rotary evaporator (Heidolph Hei-VAP Platinum 3), thus obtaining the water (DC_H2O_1) extract. The ethanolic extract was evaporated to dryness and then resuspended in 800 mL of water to obtain the ethanolic (DC_EtOH_1) extract. The removal of ethanol was necessary for further administration to the experimental animals. Finally, these concentrated extracts were further diluted with a factor of two obtaining DC_H2O_2 and DC_EtOH_2, and four times respectively, to obtain, and DC_H2O_3 and DC_EtOH_3.

### 4.4. Total Polyphenol Content (TPC)

The Folin–Ciocalteu method was used to evaluate the TPC as described in Pop et al., (2018) [50]. Accordingly, 25 mL of each plant extract sample was mixed with the following reagents: 125 mL (0.2 N) of Folin–Ciocalteu reagent and 100 mL of sodium carbonate (Na2CO3) solution (7.5% *w*/*v*). Afterward, each mixture was homogenized and further incubated at room temperature for 2 h, using dark conditions [50]. The 96-well plates were read with a Synergy HT Multi-Detection Microplate Reader (BioTek Instruments, Inc., Winooski, VT, USA) at 760 nm. The results were calculated using a gallic acid calibration curve (R^2^ = 0.9945) and further expressed as gallic acid equivalents (GAE). Analysis of each extract was performed in triplicate. Results were presented as mean values (mg/g dry weight (DW) of extracts) ± standard deviations.

### 4.5. DPPH Antioxidant Capacity Test

The DPPH radical-scavenging capacity of DC fruit extracts was evaluated using Brand-Williams adapted method [51]. Thus samples (250 μL of each extract) were mixed with 1750 μL DPPH solution (0.02 mg/mL in methanol) and further incubated for 30 min at room temperature. A 96-well plates Synergy HT Multi-Detection Microplate Reader (BioTek Instruments, Inc., Winooski, VT, U.S.A.) was used to read the absorbance at 517 nm. Methanol was used for the control. The data was reported as percentage decrease in absorbance of the sample relative to the control. The relative scavenging capacity percent of DPPH reagent was calculated using the following formula:

DPPH Scavenged (%) = ((AB–AA)/AB) × 100(1)

where, AB is absorbance of blank at t = 0 min; AA is absorbance of the sample at t = 30 min.

Analysis was performed in triplicate.

### 4.6. Fruits Ethanolic and Water Extracts Analysis

#### 4.6.1. Liquid Chromatography–Diode Array Detection–Electro-Spray Ionization Mass Spectrometry (HPLC-DAD-ESI MS)

The HPLC-MS analysis of DC water and ethanolic extracts (DC_H2O_1 and DC_EtOH_1) was performed as described in Pop et al. (2018). The equipment used was an HPLC with DAD detection (Agilent 1200) coupled to a single quadrupole mass spectrometer (Agilent 6110). The analysis was performed at room temperature using an Eclipse XDB C18 column (4.6 × 150 mm, 5 m particle size) (Agilent Technologies, Santa Clara, CA, USA) and a gradient of two mobile phases. The mobile phases were 0.1% acetic acid/acetonitrile (99:1) in distilled water (*v*/*v*) (mobile phase A) and 0.1% acetic acid in acetonitrile (*v*/*v*) (mobile phase B) [50]. A gradient of the two mobile phases was used to achieve compounds separation as follows: from 0 to 2 min (95% A), following from 2 to 18 min with a decrease from 95 to 60% A, then from 18 to 20 min a decrease from 60 to 10% A, and from 20 to 24 min 10% A. Afterwards, mobile phase A was increased again at 95% in one minute and finally kept for 5 min at 95% [50]. The separation flow rate was 0.5 mL/min. The absorbances were registered at 280 nm and 340 nm. Next, compounds were injected into the MS using the ESI source set in the (+) mode. The working temperature was 350 °C, the nitrogen flow was 8 L/min while the capillary voltage was set at 3000 V. The interval between 100 and 1000 *m*/*z* was used for compound scanning. Agilent Chem-Station Software (Rev B.04.02 SP1, Palo Alto, CA, USA) was used for data analysis. Finally, compound identification was performed by combining compounds’ UV−visible spectra, their retention time, mass spectra, and literature data.

#### 4.6.2. Fourier Transform Infrared Spectroscopy (FTIR) Analysis

The FTIR spectra of DC ethanolic and water extracts (DC_EtOH_1 and DC_H2O_1) were measured using an FTIR spectrometer (Shimadzu IR Prestige-21) equipped with attenuated total reflectance (ATR). The fruit extracts (10 mL) were pipetted directly on the Zinc Selenide Composite-ZnSe ATR crystal. The spectra were measured for a total number of 64 scans from 4000 to 650 cm^−1^.

#### 4.6.3. In-Tube Extraction Dynamic Headspace Gas-Chromatography–Mass Spectrometry (ITEX/GC-MS) Qualitative Analysis of Volatile Compounds

Sample extraction of DC_EtOH1 and DC_H2O_1 was performed using the in-tube extraction technique (ITEX) followed by compound separation and identification. Gas-chromatography–mass spectrometry (GC-MS) analysis was performed using a Shimadzu QP-2010 GC-MS model (Shimadzu Scientific Instruments, Kyoto, Japan) equipped with an autosampler (Combi-PAL AOC-5000, CTC Analytics, Zwingen, Switzerland) and a capillary column (ZB-5 ms, 30 m × 0.25 mm i.d. × 0.25 mm, Phenomenex, Torrance, CA, USA) [52]. The separation and compound identification were performed as described in Pop et al. (2020). Helium was used as carrier gas (1 mL/min) with the split ratio of 1:50. The program temperature used for compounds separation on the column oven had the following steps: 50 °C (2 min), then raised to 160 °C with 4 °C/min, then raised to 250 °C with 15 °C/min and further held for 10 min [52]. The interface temperatures, injector, and ion-source temperatures were set at 250 °C. Electron impact (EI) mode was used in the MS with ionization energy of 70 Ev. The compounds were scanned using the range between 40–500 *m*/*z* [52]. Their tentative identification was made by comparison of the mass spectrometric information with the ones from NIST27 and NIST147 mass spectra libraries. A minimum similarity of 85% was considered for each compound. The retention indices as obtained from www.flavornet.org (accessed on 15 September 2021) or www.pherobase.com (accessed on 20 September 2021) were also used.

### 4.7. Animals

In the present study, 120 female Wistar rats (150–200 g, from 10 to 12 weeks of age) were used. The animal facility at the University of Medicine and Pharmacy “Iuliu Hațieganu” Cluj-Napoca, Romania provided all the animals. During the first week of acclimatization (22–24 °C, 50–65 % humidity, 12 h/12 h light and dark cycle), animals consumed rodent standard diet and water ad libitum. Testing approaches followed the guideline referring to the principles of 3Rs (replacement, reduction, refinement). Moreover, the protocols were approved by the University of Medicine and Pharmacy “Iuliu Hațieganu” Ethics Committee and by the Sanitary-Veterinary and Food Safety Directorate from Cluj-Napoca (No. 236 from 16 November 2020).

#### 4.7.1. Experimental Design

The arthritis was induced by injecting 100 mL in the sub plantar region of each rat, in the left hind paws, except for the Sham group. Animals were randomly assigned according to their weight into 10 groups of 10 rats each (Table 5). All treatments were administered thirty minutes before CFA induction (day 0) as indicated in Table 5, and then the rats were treated daily for 30 days. The measurements were performed initially, 24 h before of CFA injection and further on day 1, day 8, day 15, day 20, and day 30. At the end of the experiment, animals were sacrificed using xylazine/ketamine overdose.

#### 4.7.2. Arthritis Assessment

Arthritis severity was evaluated considering paw volume, pain threshold, and thermal hyperalgesia at the time point previously mentioned (days 0, 1, 8, 15, 22, and 30).

#### 4.7.3. Edema Assessment 

An Ugo Basile plethysmometer (Milan, Italy) was used to measure paw edema. The plethysmometer records the volume of fluid displaced by the paw. The paw volume was considered each week (days 0, 1, 8, 15, 22, and 30).

#### 4.7.4. Pain Threshold Assessment

An Ugo Basile analgesy-meter (Milan, Italy) was used to assess the mechanical pain threshold according to the Randall–Selitto method. Linear increasing mechanical pressure was applied to the rat’s paw. Latency response was recorded until rats retracted their paw or when they squeaked. The cut-off pressure was set at 500 g.

#### 4.7.5. Thermal Hyperalgesia Assessment

An Ugo Basile hot plate (Milan, Italy) was used to evaluate thermal hyperalgesia. Heat hypersensitivity was recorded at 50 ± 0.1˚C. Each rat was placed on a hot metal plate. The nociceptive withdrawal response was recorded when the first sign of paw licking or jumping was observed. A cut-off time value of 60 s was set to prevent tissue damage [53].

#### 4.7.6. Blood Samples

Blood was collected at the end of the experiment from the retro-orbital sinus plexus. Rats were lightly anesthetized with ketamine (20 mg/kg b.w., i.p.) and xylazine (2 mg/kg b.w., i.p.). After coagulation, blood was centrifuged at 4000 rpm for 15 min; the resulting serum was stored at −80 °C for further analysis.

#### 4.7.7. ELISA Analysis

Proinflammatory cytokines TNF-a, IL-1b, IL6, and cortisol were measured using the ELISA technique (Stat Fax 303 Plus Microstrip Reader, Palm City, FL, USA). Proinflammatory cytokines detection and quantification were performed using commercially ABTS ELISA development kits (PeproTech EC, Ltd., London, UK). Cortisol was determined using a rat COR ELISA kit; (FineTest, Wuhan Fine Biotech Co., Wuhan, China). 

#### 4.7.8. Oxidative Stress Analysis

The serum oxidative stress status was evaluated by measuring malondialdehyde (MDA), nitric oxide (NO) and total thiols (THIOL), total antioxidant capacity (TAC), total oxidative status (TOS), and oxidative stability index (OSI). Malondialdehyde analysis was performed as described by Conti et al. (1991) [54]. The lipid peroxidation marker was determined using thiobarbituric acid. After an incubation of 30 min at 95 °C, the absorbances were measured at 532 nm. The results of MDA concentration were expressed in nmol/mL of serum. Next, NO analysis was performed as described by Miranda et al. (2001). Accordingly, the NO synthesis (NO*x*) assessment was performed following nitrate reduction by vanadium (III) and further detected by acidic Griess reaction [55]. Total thiol analysis was performed as described by Hu et al. (1994). Accordingly, the reaction with Ellman’s reagent (2,2-dithiobisnitrobenzoic acid or DTNB) was used [56]. Total antioxidant capacity was determined using the method described by Erel (2004), which is based on potent free radical reactions occurring due to the initiation of hydroxyl radical (OH) production via Fenton reaction [57]. Total oxidative status analysis was performed using a colorimetric method that quantifies the oxidation of ferrous ions to ferric ions [58]. Finally, OSI was calculated using the ratio between TOS and TAC values [59].

### 4.8. Statistical Analysis

The statistical analysis was performed using GraphPad InStat 3.1 software (San Diego, CA, USA). Mean ± Standard Error of Mean (SEM) was used to express the results of the study. For statistical analysis, multiple comparisons of data were carried out using two and one-way analysis of variance (ANOVA), and then post-hoc test of Tukey was used. Significance was statistically acceptable at a level of *p* < 0.05.

## 5. Conclusions

The results of this study showed that both water and ethanolic DC extracts contained bioactive compounds, mainly flavan-3-ols, proanthocyanidins, and flavone glycosides, responsible for the in vitro antioxidant capacity. The in vivo analgesic and anti-inflammatory activity evaluated by measuring the antinociceptive effect and paw volume was maximum after 1 day from CFA injection and showed promising results in the long term as well. The antioxidant and anti-inflammatory effects assessed by measuring the pro-inflammatory cytokines, cortisol, and oxidative stress parameters showed that DC administration significantly reduced these parameters, but their effect was dose and extract type-dependent. Some of the DC extract results were like those of diclofenac. These results suggest the need for further studies in order to develop a standardized extract with an optimum concentration.

## Figures and Tables

**Table 1:** Total polyphenols content and relative 2,2-diphenyl1-picrylhydrazyl (DPPH) radical scavenging capacity of *Dichrostachys cinerea* fruits water and ethanolic extracts.

Extracts	Total Polyphenols Content (mg GAE/g Dry Weight Fruits)	Radical Scavenging Capacity of DC Extracts by DPPH (Relative %)
DC_H2O_1	153.75 ± 7.16	38.29 ± 0.41
DC_H2O_2	74.90 ± 4.66	23.60 ± 0.32
DC_H2O_3	39.04 ± 3.11	13.19 ± 0.22
DC_EtOH_1	207.19 ± 4.28	72.41 ± 0.50
DC_EtOH_2	104.50 ± 7.06	39.53 ± 0.39
DC_EtOH_3	52.68 ± 5.13	20.37 ± 0.45

The results are expressed as mean ± standard deviation. GAE—gallic acid equivalents; DPPH-2,2-diphenyl1-picrylhydrazyl.

**Table 2:** Liquid Chromatography–Diode Array Detection–Electro-Spray Ionization Mass Spectrometry phenolic compound tentative identification from *Dichrostachys cinerea* fruits water and ethanolic extracts.

No	Rt (min)	Lambda Max (nm)	M/ [M+H]^+^ *^m^*^/*z*^	Tentative Identification	Extract Type DC_H2O_1 (W) DC_EtOH_1 (E)	Compound Class	References
1	11.6	280	579	Procyanidin dimer (C-C)	W, E	Proanthocyanidin	[20]
2	12.9	280	291	Catechin	W, E	Flavan-3-ol	[20]
3	13.3	280	579	Procyanidin dimer (C-EC)	W, E	Proanthocyanidin	[20]
4	14.1	280	291	Epicatechin	W, E	Flavan-3-ol	[20]
5	14.4	sh 240, 275, 340	565	Apigenin-8-C-glucoside-2’-Oxyloside	W, E	Flavone glicoside	[21]
6	14.8	280	731	ECG-EC Procyanidin Dimer	W, E	Proanthocyanidin	[22]
7	15.7	280	715	EGC-EC Procyanidin Dimer	W, E	Proanthocyanidin	[22]
8	16.4	280	443	Catechin-gallate	W, E	Flavan-3-ol	[20]
9	17.0	280	443	Epicatechin-gallate	W	Flavan-3-ol	[20]
10	17.7	260, 350	449	Quercetin-rhamnoside	W, E	Flavone glycosides	[23]
11	18.2	225, 300	535	Resveratrol derivative	W, E	Stilbenoids	[24]
12	19.5	235, 285, sh 310, 380	419	Kaempferol 7-arabinoside	W, E	Flavone glicoside	[24,25]
13	20.1	235, 280 sh 315	255	Daidzein	E	Isoflavone	[26]
14	20.8	sh 210, 230, 285, 315	437	Catechin rhamnoside	E	Flavan-3-ol glycoside	[27]
15	22.9	240, 280	317	(Iso)Rhamnetin	E	Flavonol	[28]
16	24.7	sh 310, 350	287	Kaempferol	E	Flavonol	[28]
17	28.8	280	1122	Proanthocyanidin-tetramer	E	Proanthocyanidin	[29]

**Table 3:** Volatile compounds composition of *Dichrostachys cinerea* ethanolic (DC_EtOH_1) extract identified by GC-MS.

Compounds	Rt (min)	Concentration (% of Total Peak Area)
Ethanedioic acid, dimethyl ester	4.723	16.19
Butanedioic acid, dimethyl ester	11.493	1.94
Benzoic acid, methyl ester	13.89	8.97
Dimethyl malate	14.944	72.89

**Table 4:** Effect of *Dichrostachys cinerea* fruit extracts on oxidative stress parameters serum levels in CFA-induced arthritic rats.

GROUPS	TOS	OSI	NOx	MDA	TIOLS
SHAM	3.58 ± 0.76 ^b^	3.29 ± 0.70 ^b^	22.72 ± 3.58 ^b^	2.78 ± 1.08 ^b^	417.20 ± 31.29 ^b^
CTRL	8.16 ± 1.17 ^a^	7.51 ± 1.07 ^a^	52.71 ± 7.93 ^a^	5.37 ± 0.87 ^a^	275.75 ± 67.74 ^a^
CTRL + DIC	6.90 ± 0.83 ^a^	6.33 ± 0.77 ^a^	28.08 ± 3.41 ^b^	3.70 ± 0.15	318.60 ± 120.58 ^a^
CTRL + DIA	6.11 ± 1.36 ^a^	5.61 ± 1.25 ^a^	28.77 ± 4.46 ^a,b^	2.63 ± 1.52 ^b^	316.40 ± 53.85 ^a^
DC_EtOH_1	6.08 ± 1.41 ^a^	5.59 ± 1.29 ^a^	29.91 ± 3.74 ^a,b^	3.51 ± 0.35 ^b^	339.00 ± 28.02
DC_EtOH_2	6.59 ± 1.91 ^a^	6.05 ± 1.75 ^a^	26.74 ± 1.57 ^b^	3.15 ± 0.42 ^b^	360.00 ± 56.13 ^a^
DC_EtOH_3	5.36 ± 0.80 ^a,b^	4.92 ± 0.74 ^a,b^	32.36 ± 4.26 ^a,b^	3.19 ± 1.33 ^b^	315.25 ± 68.14 ^a^
DC_H2O_1	5.70 ± 1.29 ^a,b^	5.23 ± 1.19 ^a,b^	36.28 ± 5.56 ^a,b^	3.30 ± 1.37 ^b^	346.00 ± 70.29 ^a^
DC_H2O_2	6.84 ± 0.60 ^a^	6.29 ± 0.55 ^a^	33.66 ± 3.83 ^a,b^	3.75 ± 0.36 ^b^	322.43 ± 44.03 ^a^
DC_H2O_3	5.41 ± 1.89 ^a,b^	4.98 ± 1.74 ^a,b^	31.87 ± 6.14 ^a,b^	3.47 ± 0.54 ^b^	300.75 ± 47.89 ^a^

Values are expressed as mean ± standard error of the mean (n = 10). Superscript ‘^a^’ indicates a significant difference (*p* < 0.05) as compared to the SHAM group while superscript ‘^b^’ indicates a significant difference (*p* < 0.05) as compared to the CTRL group as performed by one-way ANOVA, Tukey’s multiple comparisons test (TOS); total oxidative status; (OSI): oxidative stability index; (NOx): nitric oxide synthesis; (MDA): malondialdehyde; (THIOL): total thiols.

**Table 5:** Experimental design of CFA-induced chronic inflammation.

Groups/Abbrev.	Administrated Substance/Dose	Route of Administration
SHAM	Normal saline solution	p.o.
Control (CTRL)	Normal saline solution + CFA	p.o.
Positive control		
(CTRL_DIC)	Diclofenac sodium (7.5 mg/kg) + CFA	i.p.
Positive control		
(CTRL_DIA)	Diazepam (0.5 mg/kg) + CFA	i.p.
Treatment		
(DC_EtOH_1)	DC_EtOH_1 (0.5 mL/100 g out of conc 1–207 mg GAE /g) + CFA	p.o.
Treatment		
(DC_EtOH_2)	DC_EtOH_2 + (0.5 mL/100 g out of conc 2–105 mg GAE/g) + CFA	p.o.
Treatment		
(DC_EtOH_3)	DC_EtOH_3 + (0.5 mL/100 g out of conc 3–53 mg GAE/g) + CFA	p.o.
Treatment		
(DC_H2O_1)	DC_H2O_1 (0.5 mL/100 g out of conc 1–154 mg GAE/g) + CFA	p.o.
Treatment		
(DC_H2O_2)	DC_H2O_2 (0.5 mL/100 g out of conc 1–75 mg GAE/g) + CFA	p.o.
Treatment		
(DC_H2O_3)	DC_H2O_3 (0.5 mL/100 g out of conc 1–39 mg GAE/g) + CFA	p.o.

Abbreviations: CFA, complete Freund adjuvant, p.o.: oral administration by gavage, i.p.: intraperitoneal injection.

## Data Availability

Not applicable.

## Supplementary Materials

The following supporting information can be downloaded at: https://www.mdpi.com/article/10.3390/molecules27175445/s1, Figures S1–S38: LC chromatograms and UV-Vis and MS spectra of compounds identified in *Dichrostachys cinerea* fruits water and ethanolic extracts.

## Sample Availability

Samples of the compounds are available from the authors.
