# Supplementary material for: Polyphenols from Dichrostachys cinerea Fruits Anti-Inflammatory, Analgesic, and Antioxidant Capacity in Freund’s Adjuvant-Induced Arthritic Rat Model
Source: Molecules. 2022 Aug 25;27(17):5445. doi: 10.3390/molecules27175445 (PMC9457916; doi:10.3390/molecules27175445)
Supplement: Supplementary file 1 [file molecules-27-05445-s001.zip › molecules-1853533-supplementary.pdf]

Supplementary file S1

LC chromatograms and UV-Vis and MS spectra of compounds identified in D*ichrostachys cinerea* fruits water and ethanolic extracts

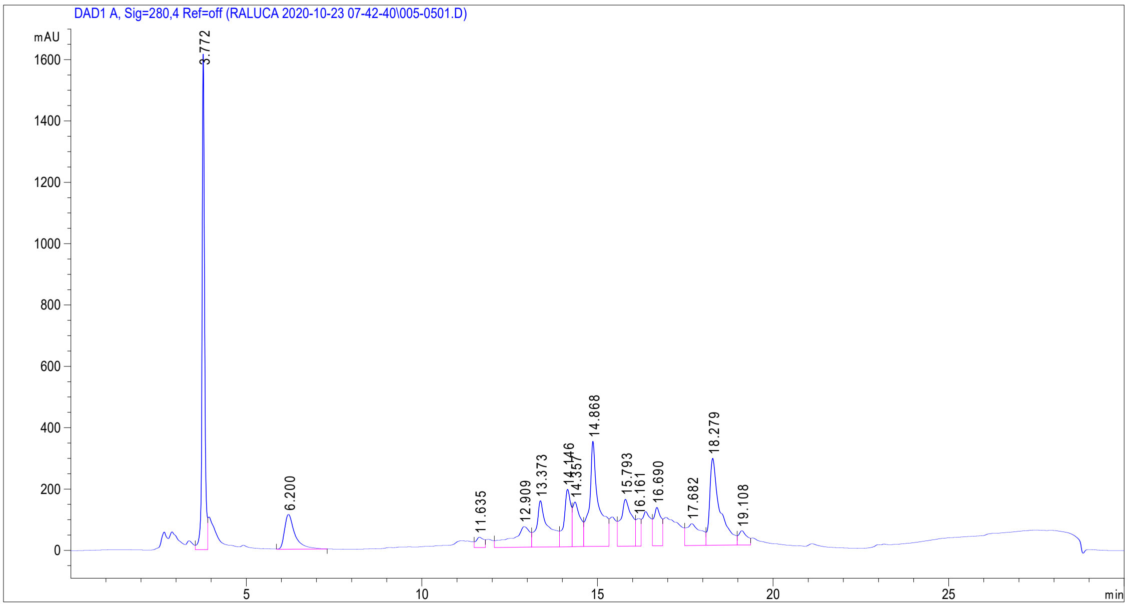

Figure S1. LC chromatogram of *Dichrostachys cinerea* water extract (DC_H2O_1 (W)) diluted 10 times at 280 nm

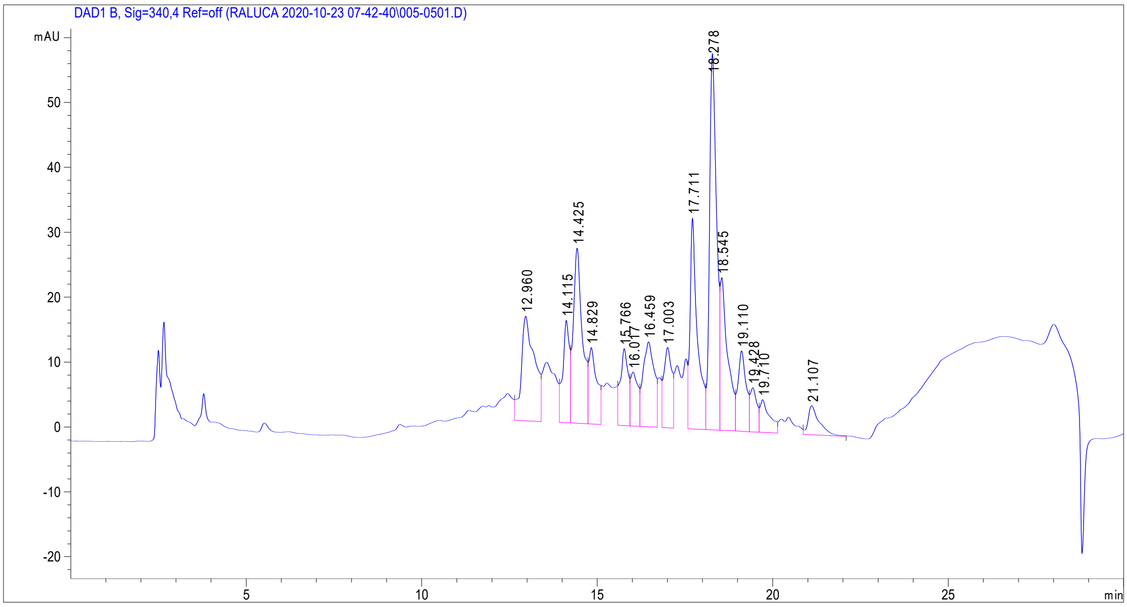

Figure S2. LC chromatogram of *Dichrostachys cinerea* water extract (DC_H2O_1 (W)) diluted 10 times at 340 nm

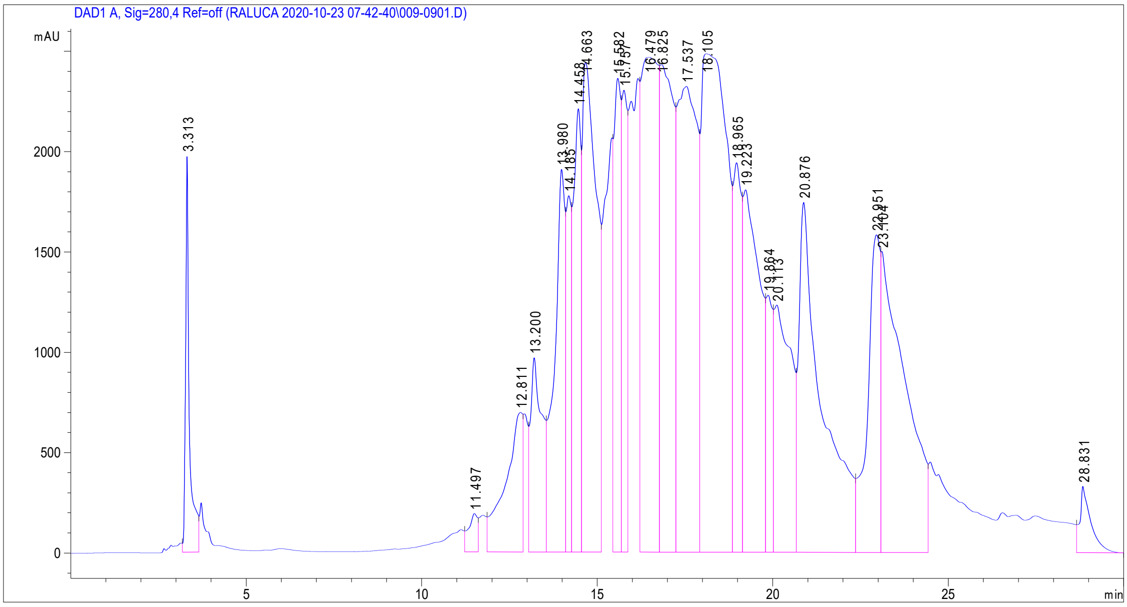

Figure S3. LC chromatogram of *Dichrostachys cinerea* ethanol extract (DC_EtOH_1 (E)) diluted 10 times at 280 nm

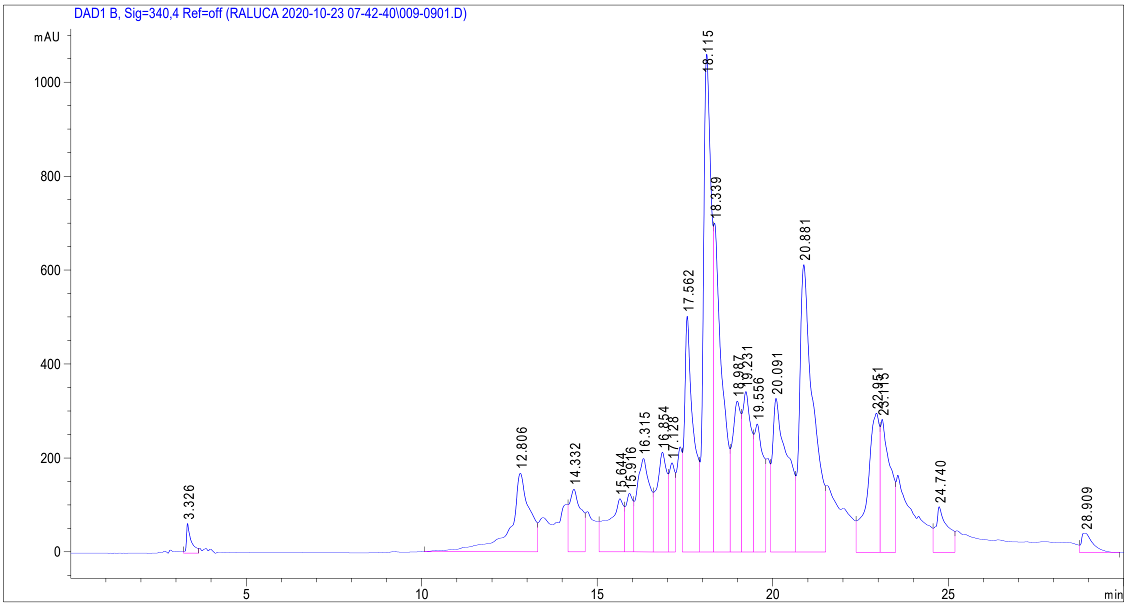

Figure S4. LC chromatogram of *Dichrostachys cinerea* ethanol extract (DC_EtOH_1 (E)) diluted 10 times at 340 nm

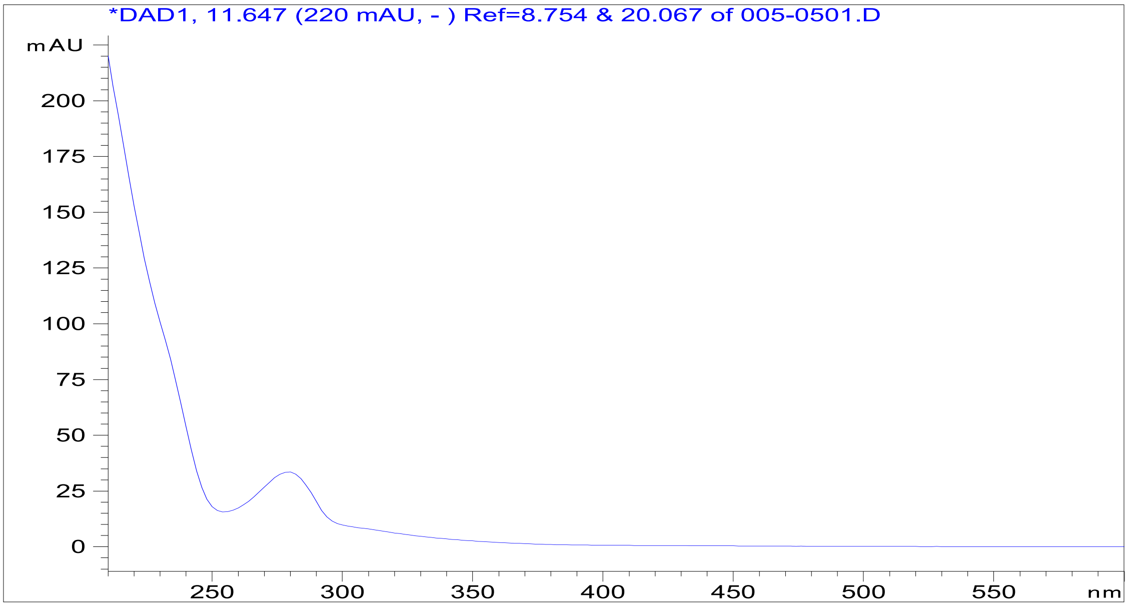

Figure S5. UV-Vis spectrum of compound No1 (Table 1) eluted at Rt 11.6 min, identified as Procyanidin dimer (C-C)

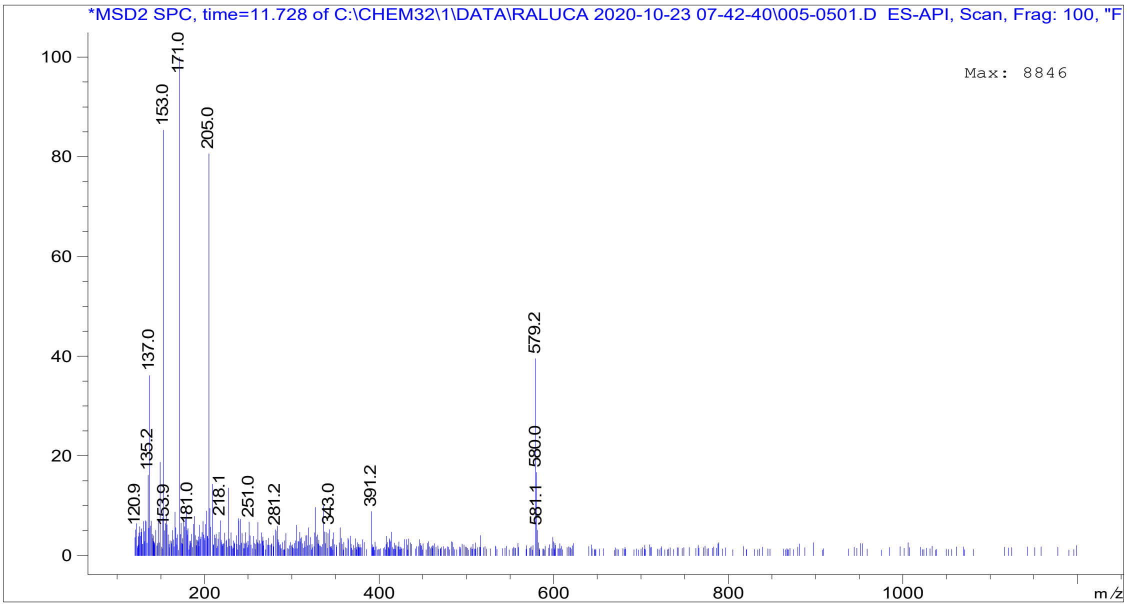

Figure S6. MS spectrum of compound No1 (Table 1) eluted at Rt 11.6 min, identified as Procyanidin dimer (C-C)

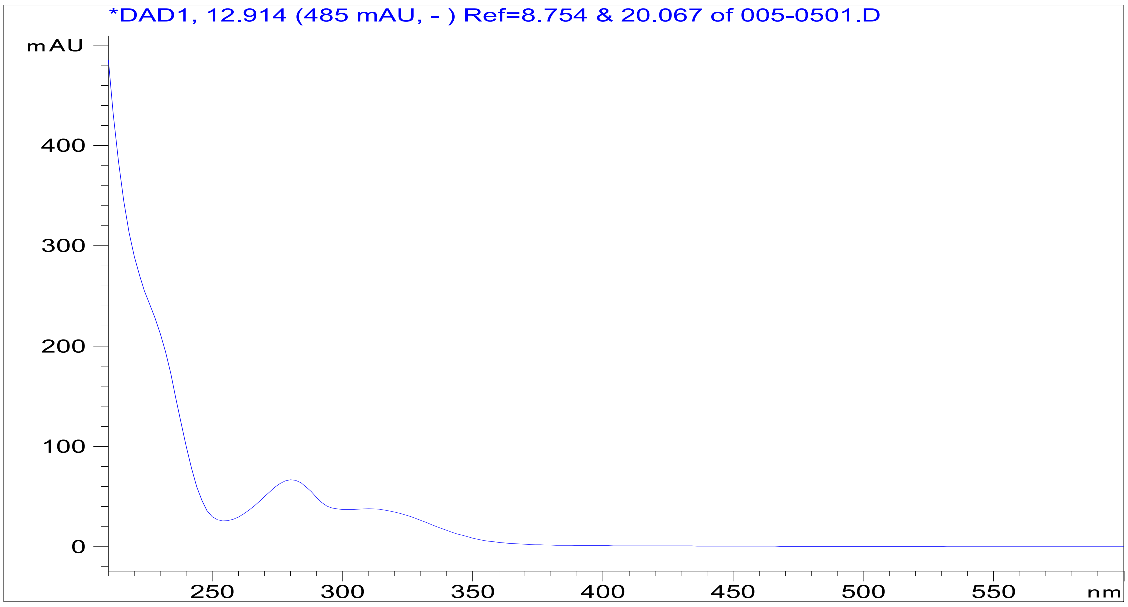

Figure S7. UV-Vis spectrum of compound No2 (Table 1) eluted at Rt 12.9 min, identified as Catechin

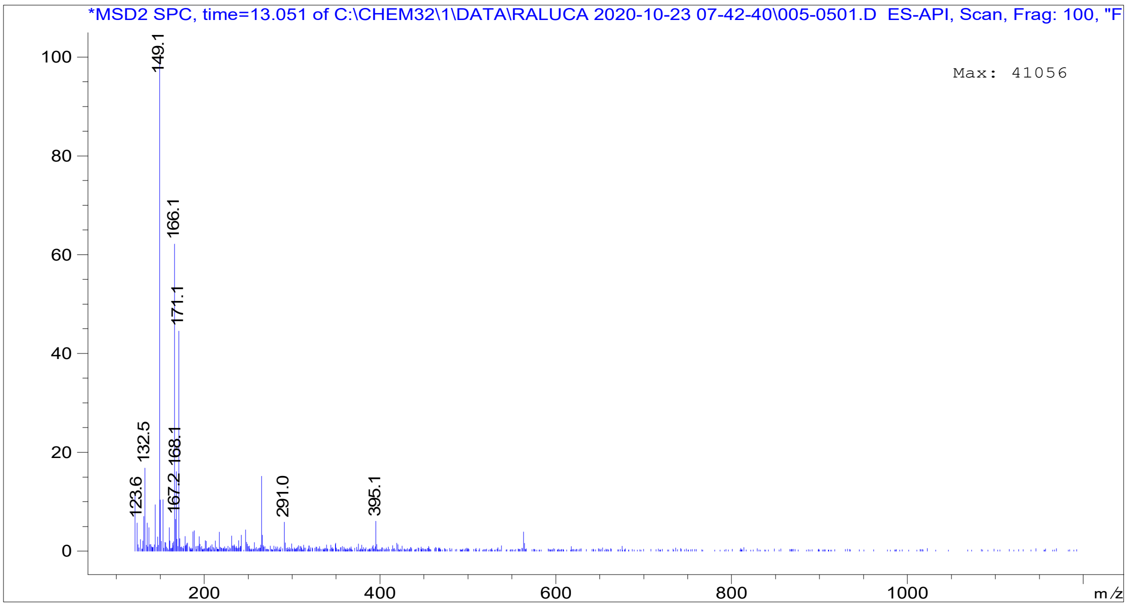

Figure S8. MS spectrum of compound No2 (Table 1) eluted at Rt 12.9 min, identified as Catechin

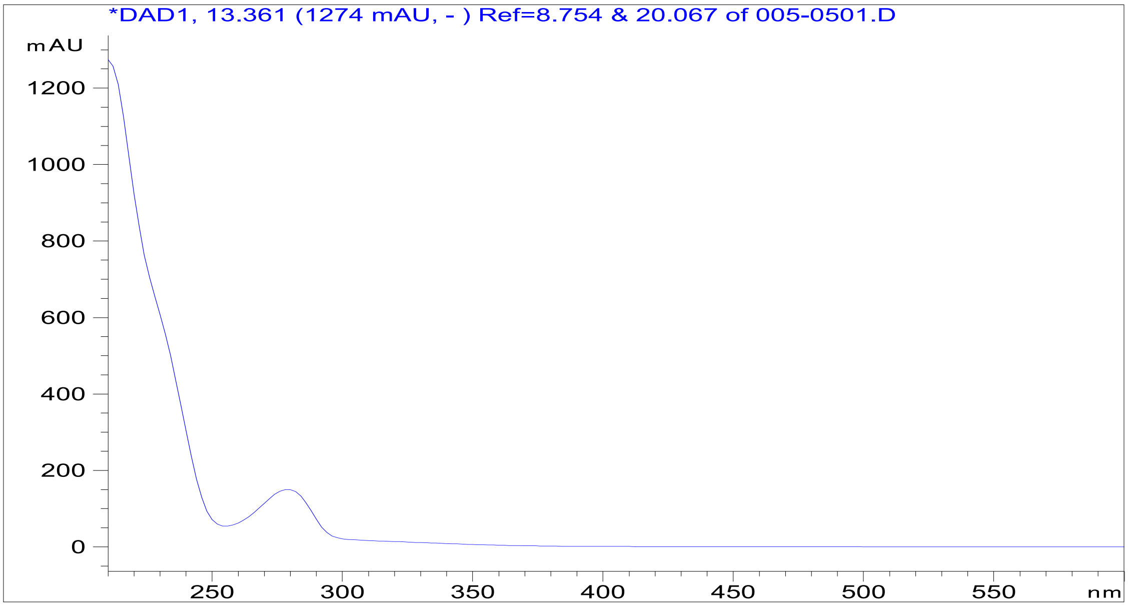

Figure S9. UV-Vis spectrum of compound No3 (Table 1) eluted at Rt 13.3 min, identified as Procyanidin dimer (C-EC)

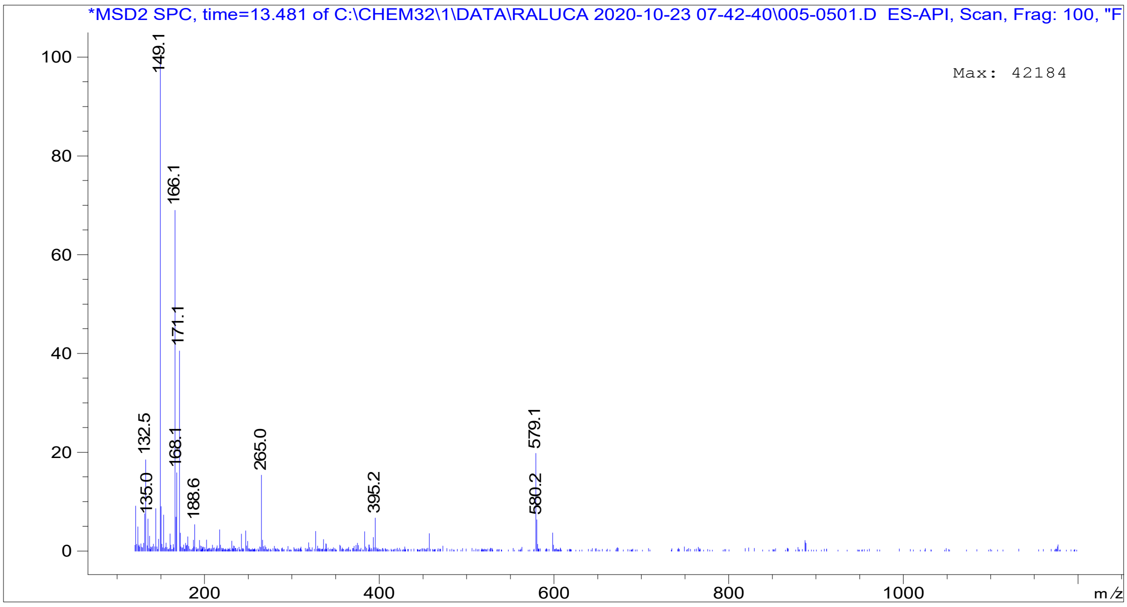

Figure S10. MS spectrum of compound No3 (Table 1) eluted at Rt 13.3 min, identified as Procyanidin dimer (C-EC)

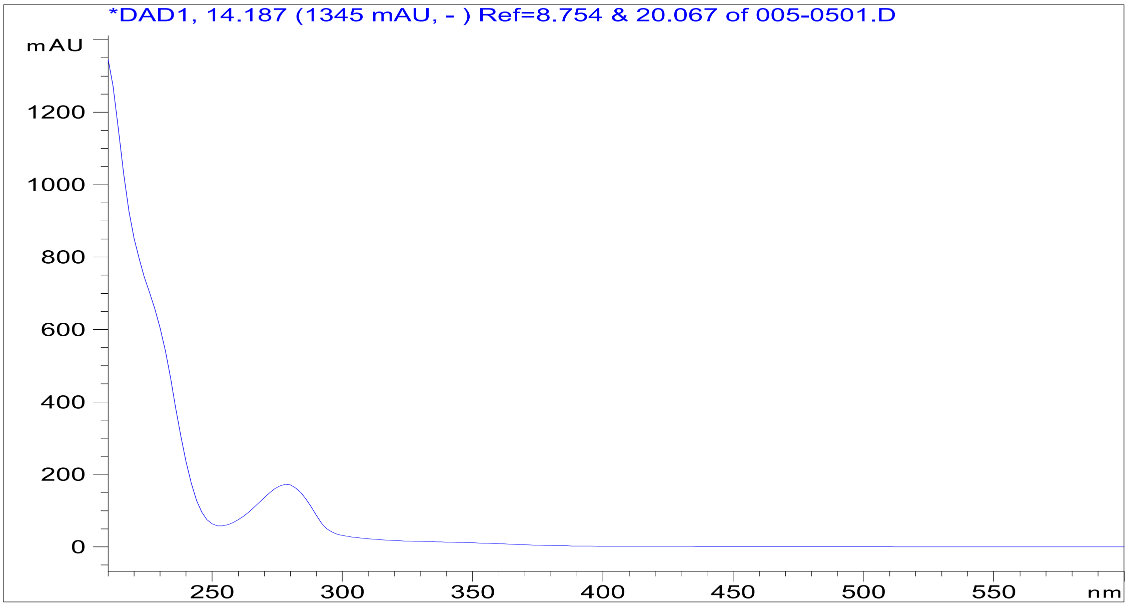

Figure S11. UV-Vis spectrum of compound No 4 (Table 1) eluted at Rt 14.1 min, identified as Epicatechin

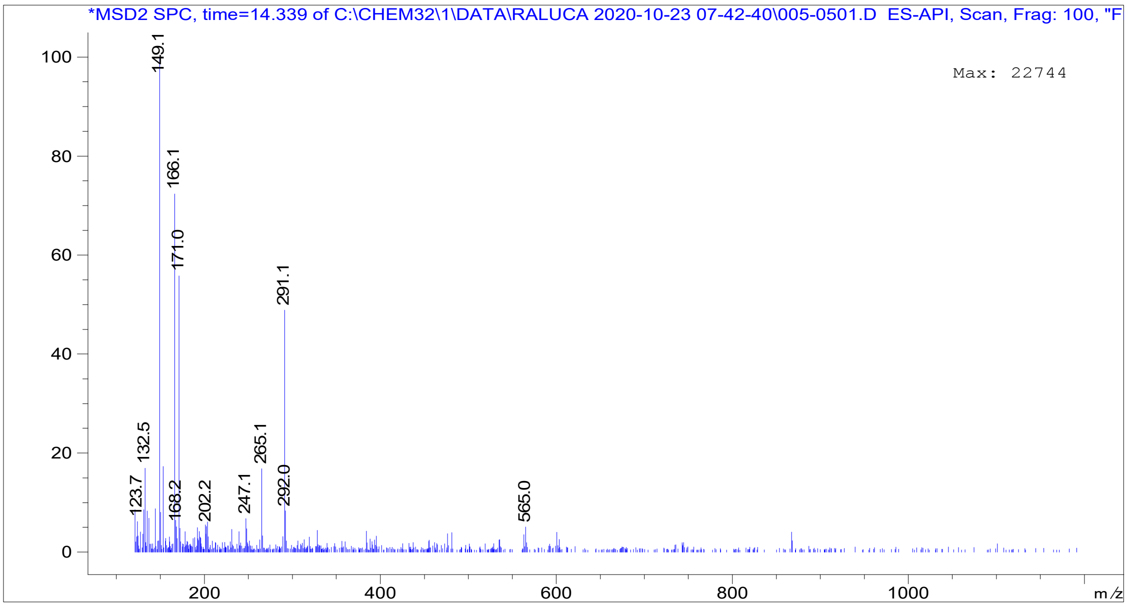

Figure S12. MS spectrum of compound No 4 (Table 1) eluted at Rt 14.1 min, identified as Epicatechin

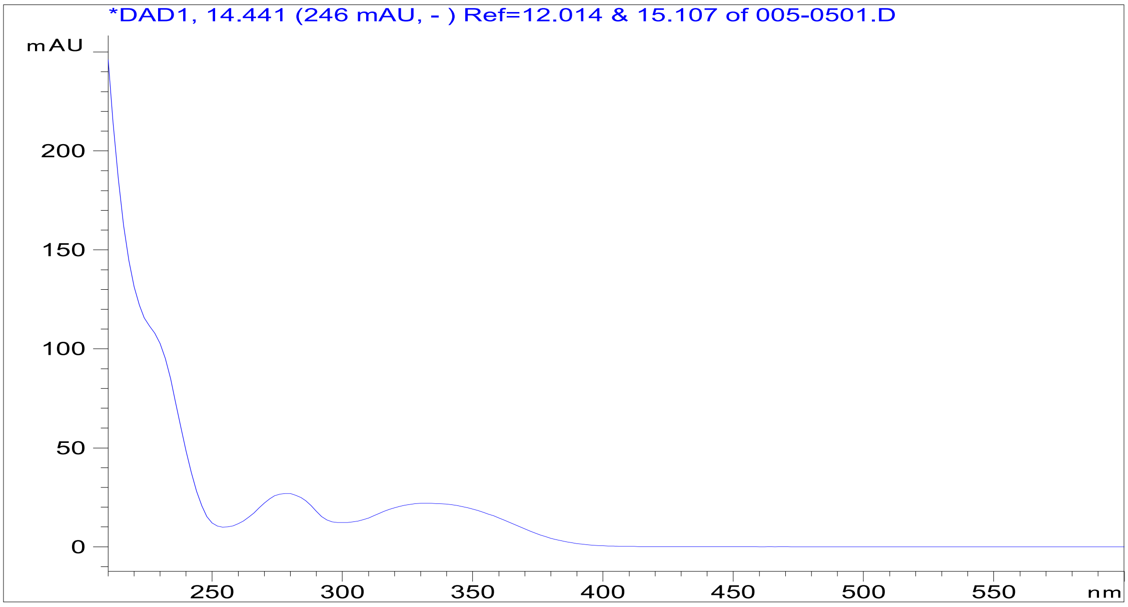

Figure S13. UV-Vis spectrum of compound No 5 (Table 1) eluted at Rt 14.4 min, identified as Apigenin-8-C-glucoside-2′′-Oxyloside

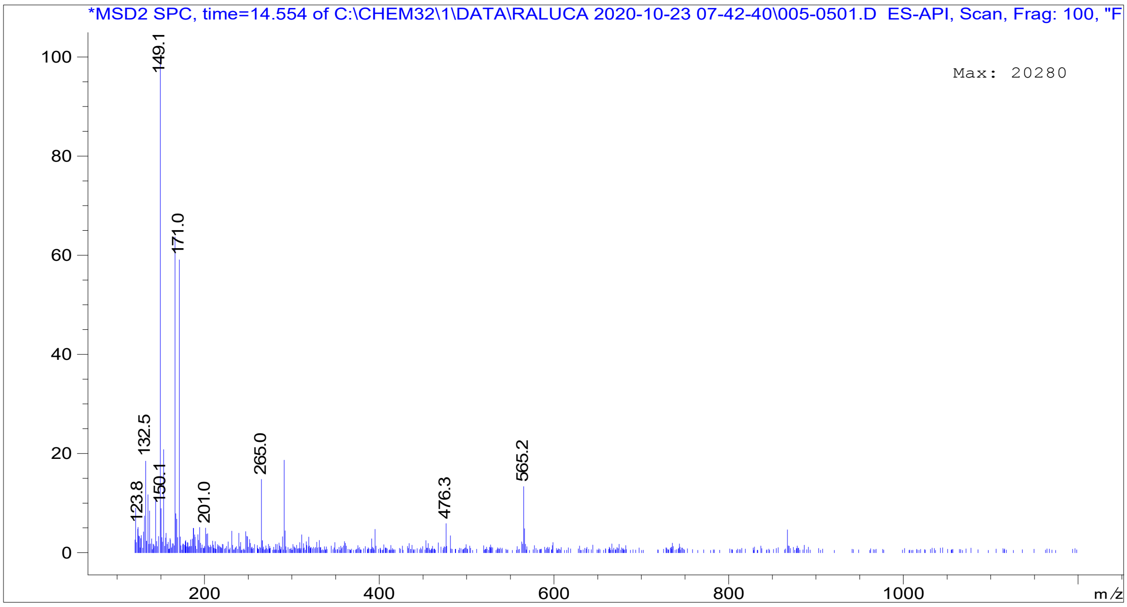

Figure S14. MS spectrum of compound No 5 (Table 1) eluted at Rt 14.4 min, identified as Apigenin-8-C-glucoside-2′′-Oxyloside

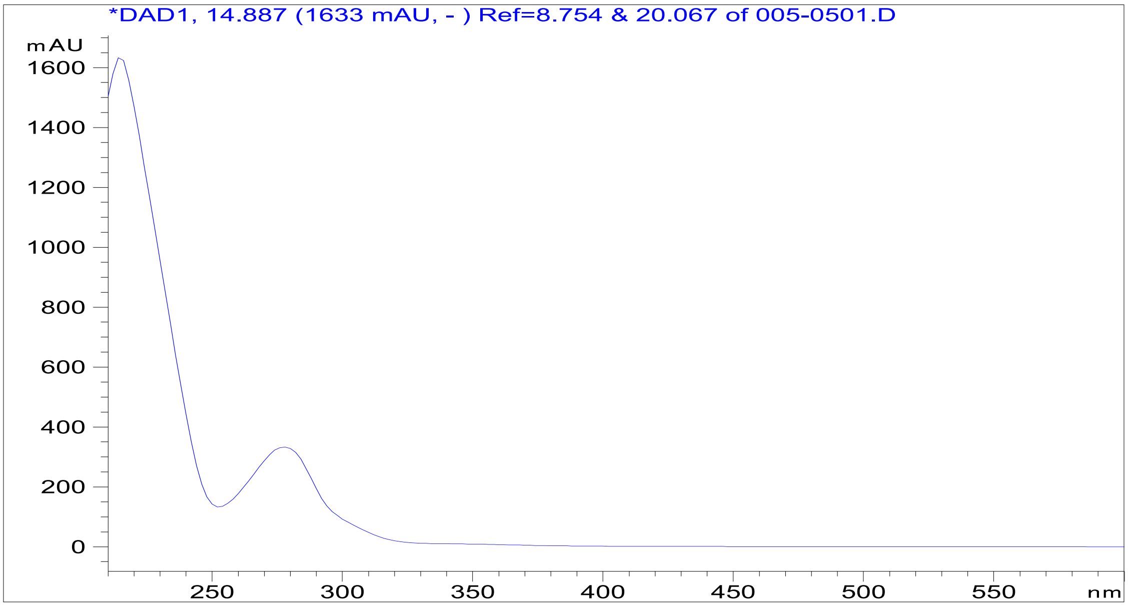

Figure S15. UV-Vis spectrum of compound No 6 (Table 1) eluted at Rt 14.8 min, identified as ECG-EC Procyanidin Dimer

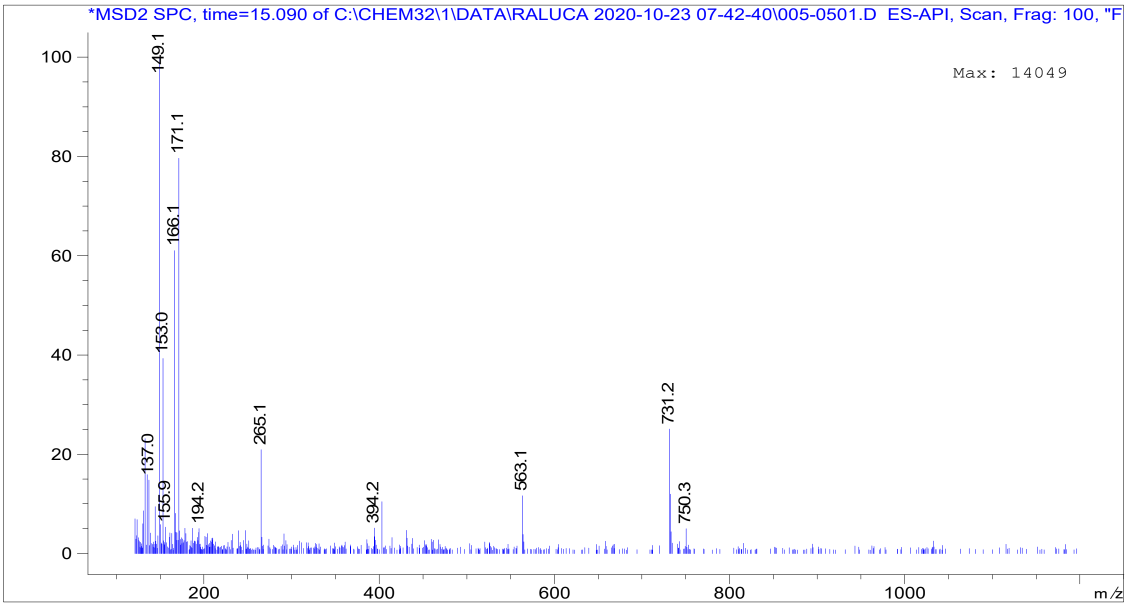

Figure S16. MS spectrum of compound No 6 (Table 1) eluted at Rt 14.8 min, identified as ECG-EC Procyanidin Dimer

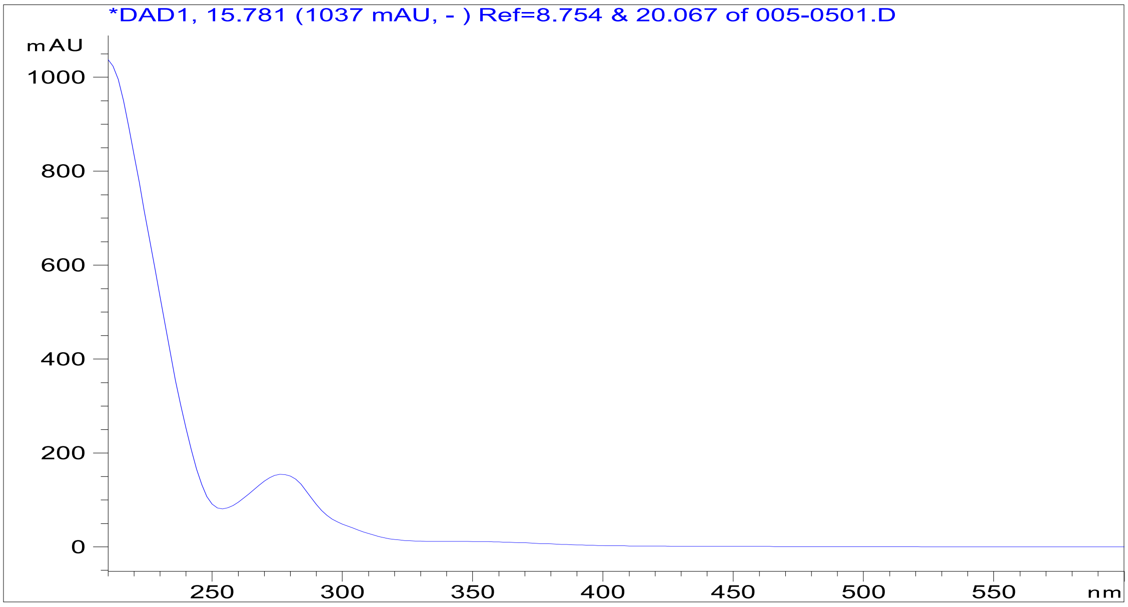

Figure S17. UV-Vis spectrum of compound No 7 (Table 1) eluted at Rt 15.7 min, identified as EGC-EC Procyanidin Dimer

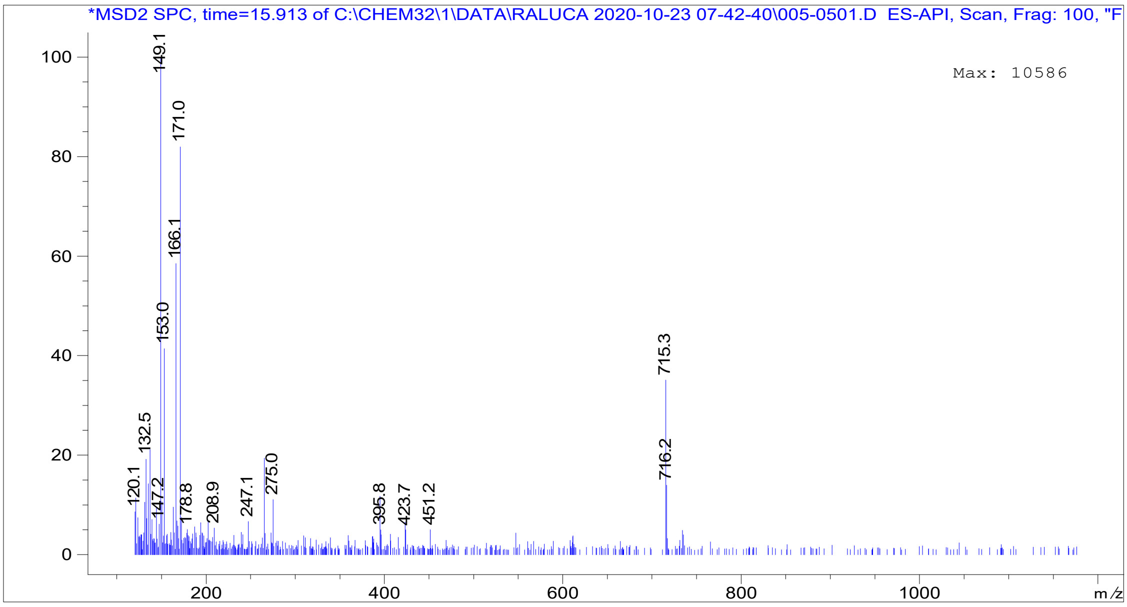

Figure S18. UV-Vis spectrum of compound No 7 (Table 1) eluted at Rt 15.7 min, identified as EGC-EC Procyanidin Dimer

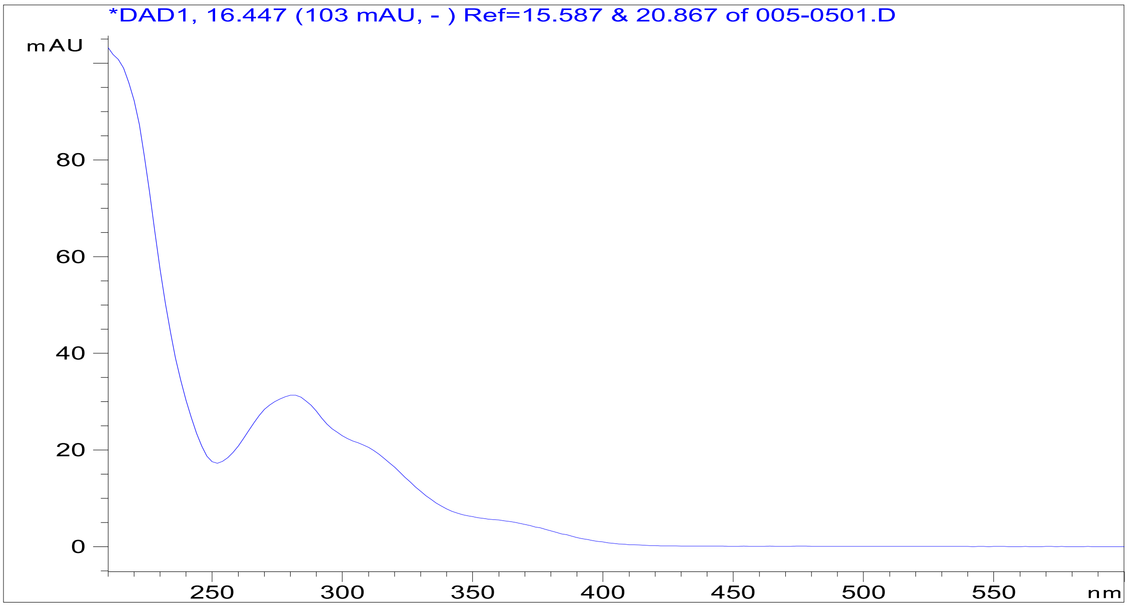

Figure S19. UV-Vis spectrum of compound No 8 (Table 1) eluted at Rt 16.4 min, identified as Catechin-gallate

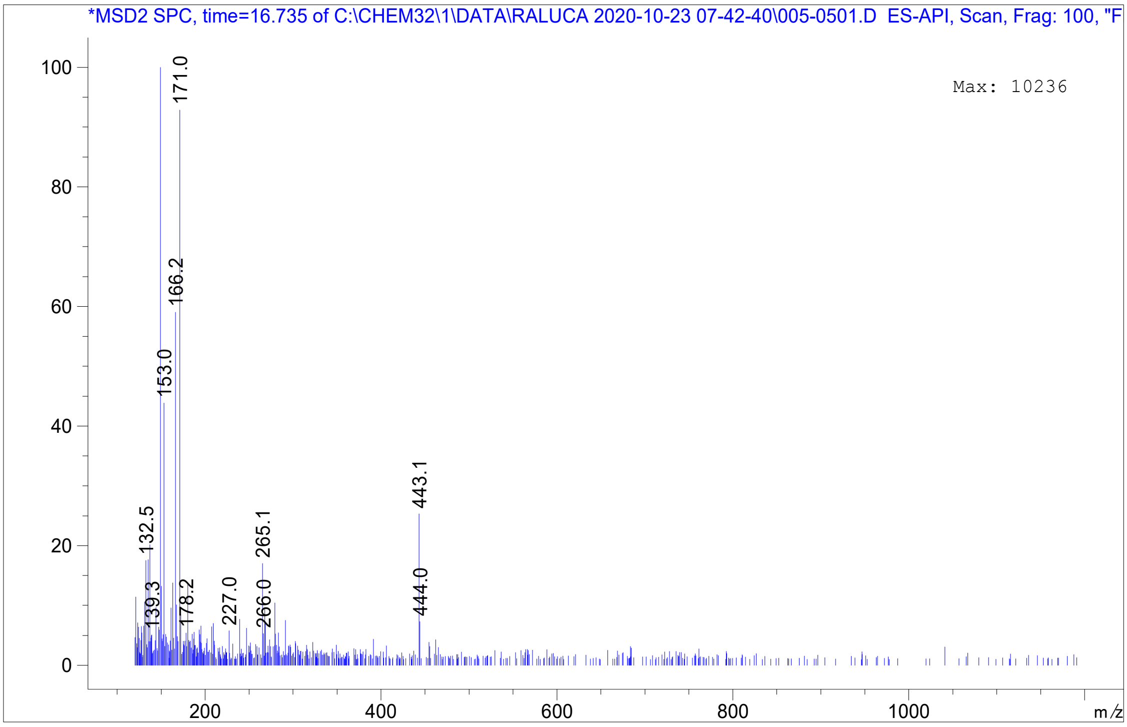

Figure S20. MS spectrum of compound No 8 (Table 1) eluted at Rt 16.4 min, identified as Catechin-gallate

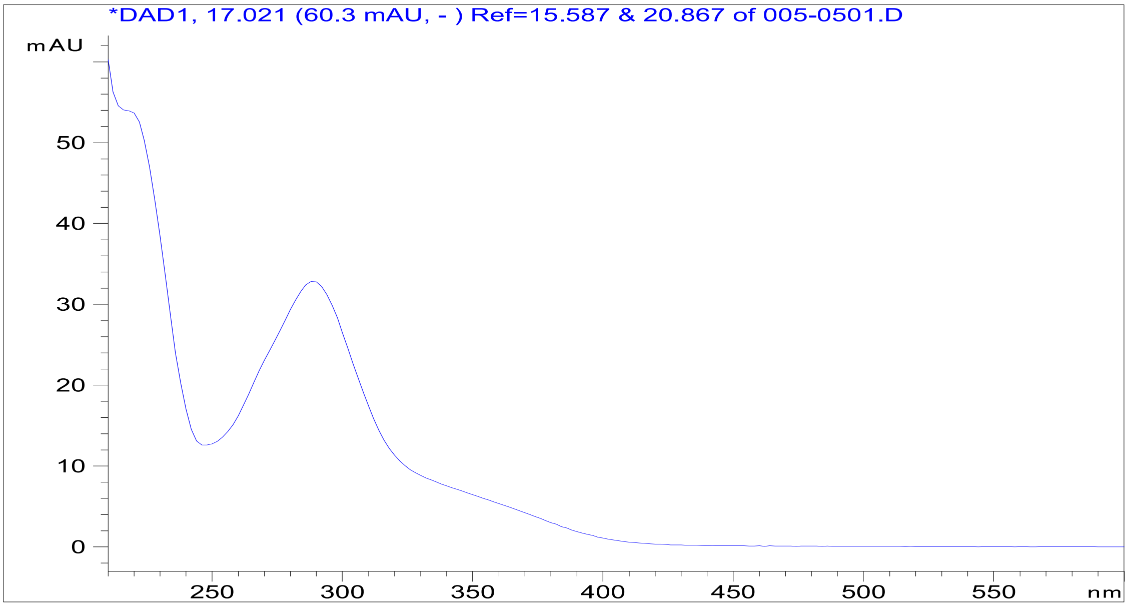

Figure S21. UV-Vis spectrum of compound No 9 (Table 1) eluted at Rt 17.0 min, identified as Epicatechin-gallate

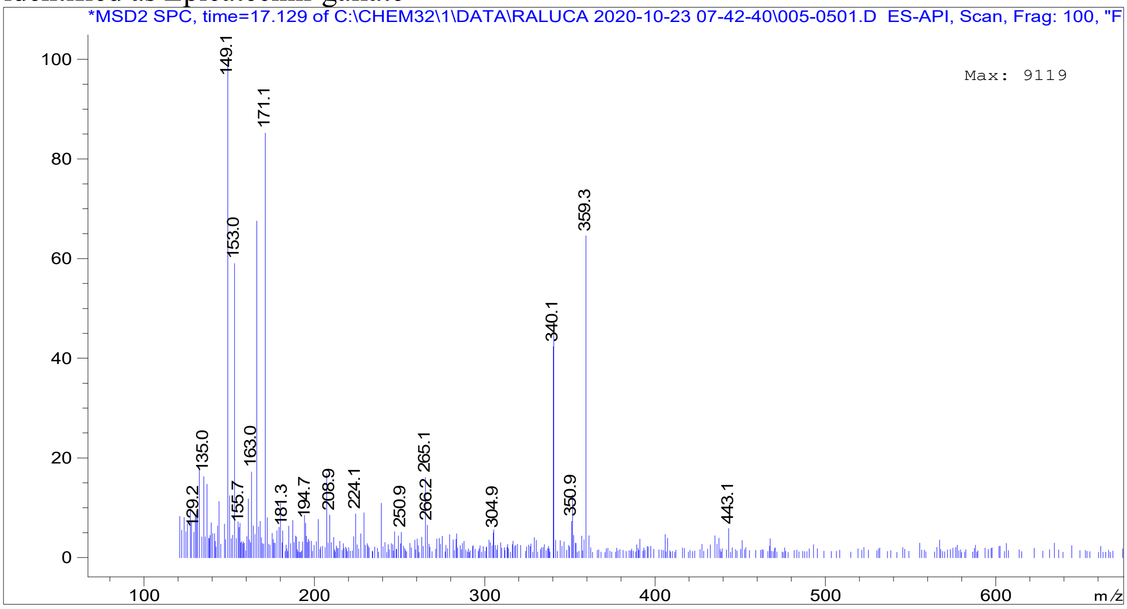

Figure S22. MS spectrum of compound No 9 (Table 1) eluted at Rt 17.0 min, identified as Epicatechin-gallate

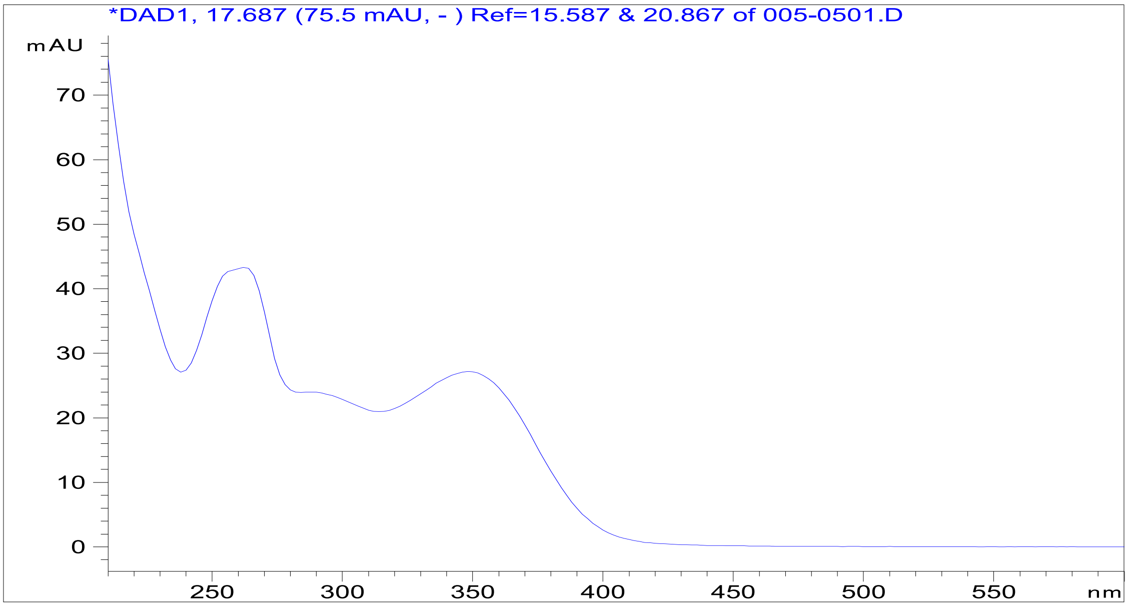

Figure S23. UV-Vis spectrum of compound No 10 (Table 1) eluted at Rt 17.7 min, identified as Quercetin-rhamnoside

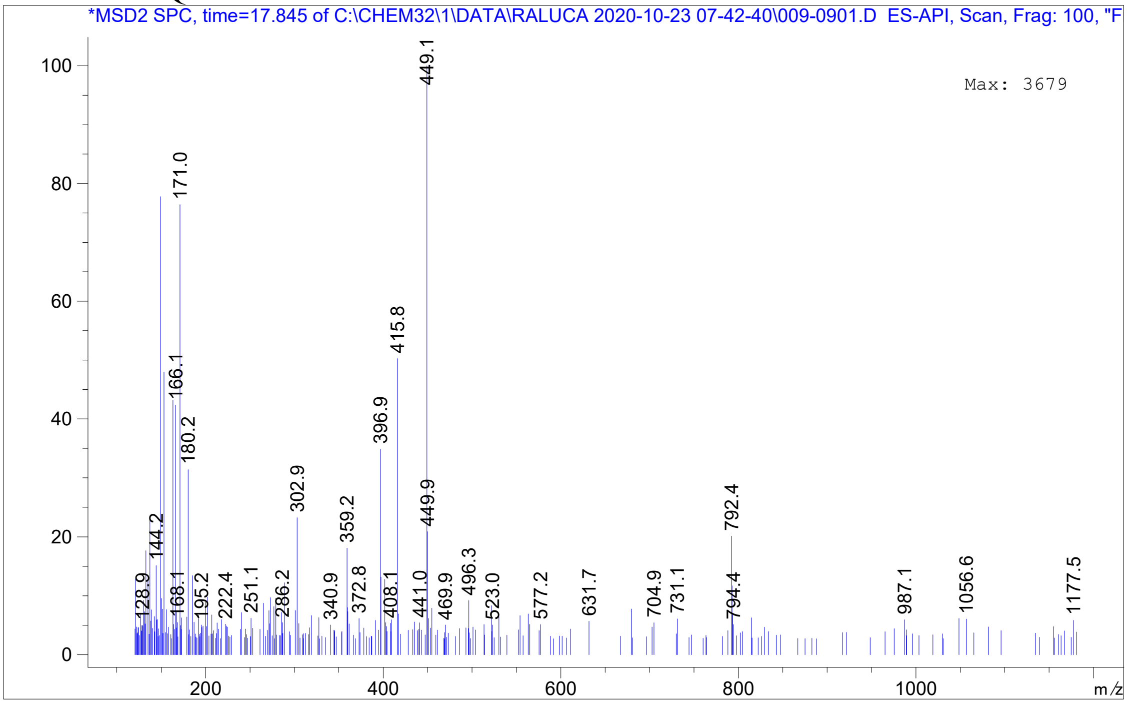

Figure S24. MS spectrum of compound No 10 (Table 1) eluted at Rt 17.7 min, identified as Quercetin-rhamnoside

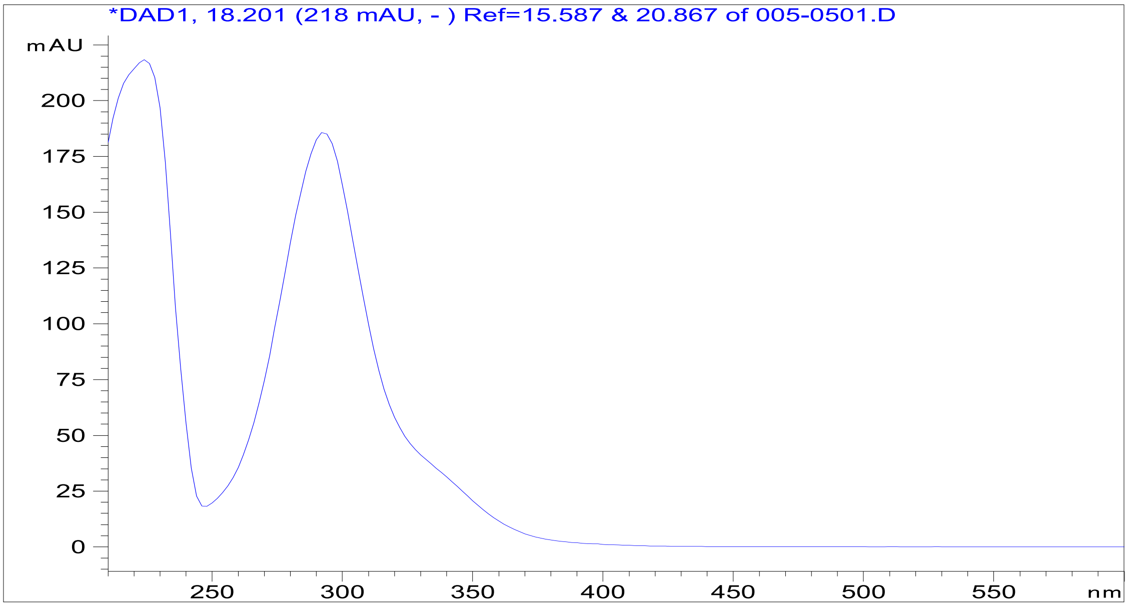

Figure S25. UV-Vis spectrum of compound No 11 (Table 1) eluted at Rt 18.2 min, identified as Resveratrol derivative

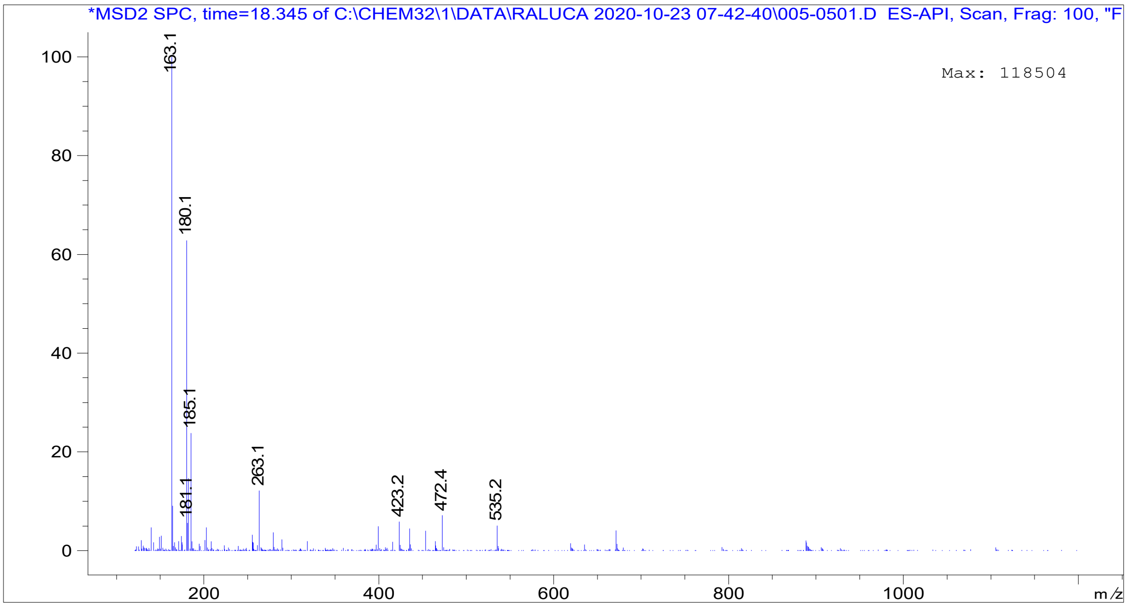

Figure S26. MS spectrum of compound No 11 (Table 1) eluted at Rt 18.2 min, identified as Resveratrol derivative

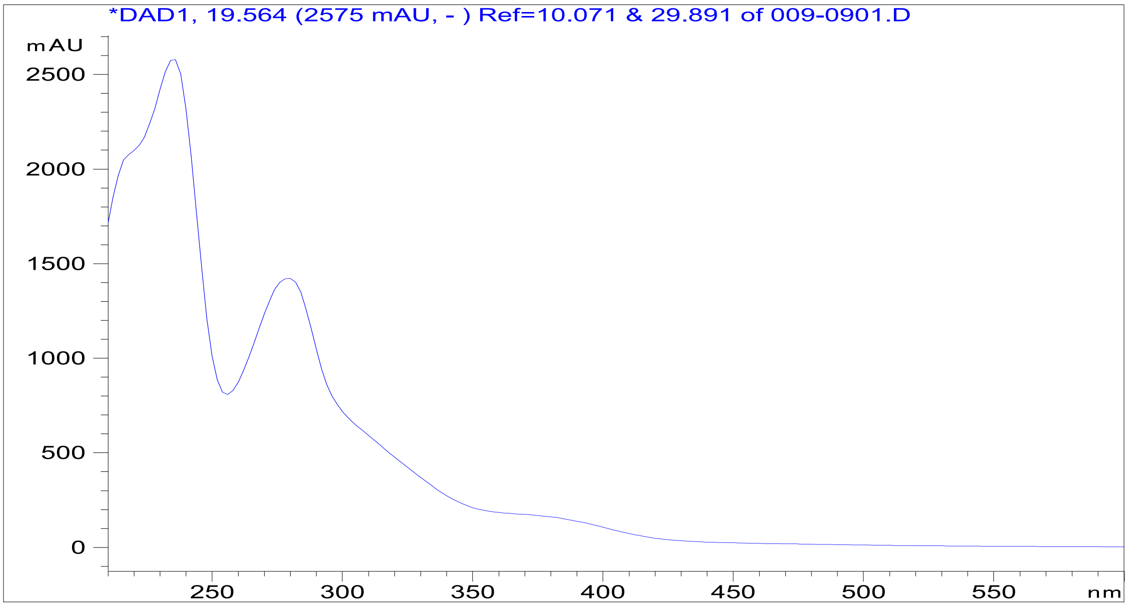

Figure S27. UV-Vis spectrum of compound No 12 (Table 1) eluted at Rt 19.5 min, identified as Kaempferol 7-arabinoside

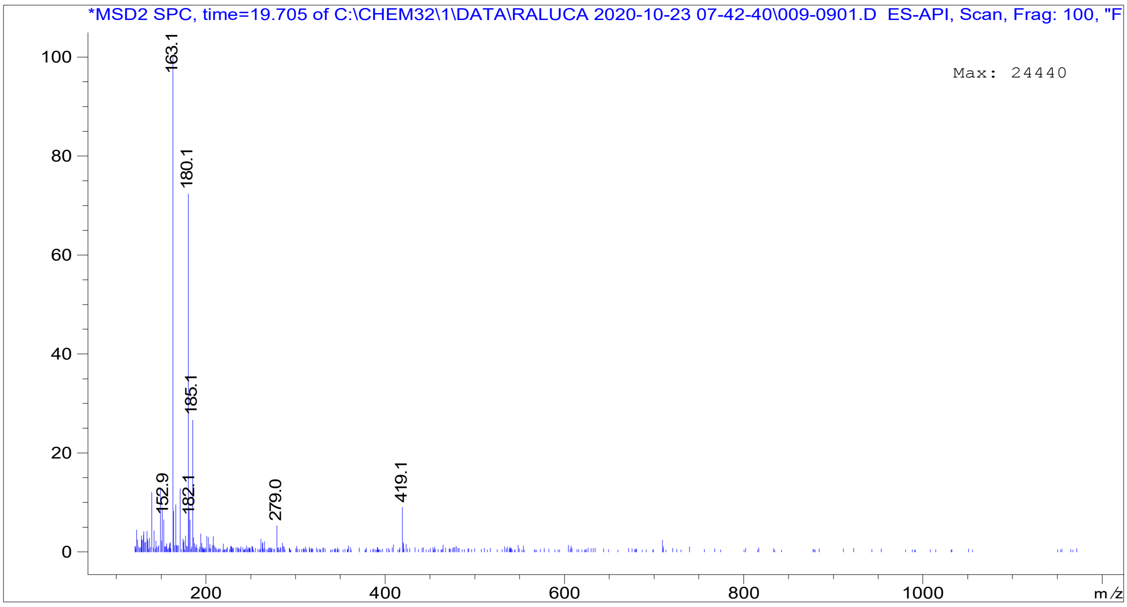

Figure S28. MS spectrum of compound No 12 (Table 1) eluted at Rt 19.5 min, identified as Kaempferol 7-arabinoside

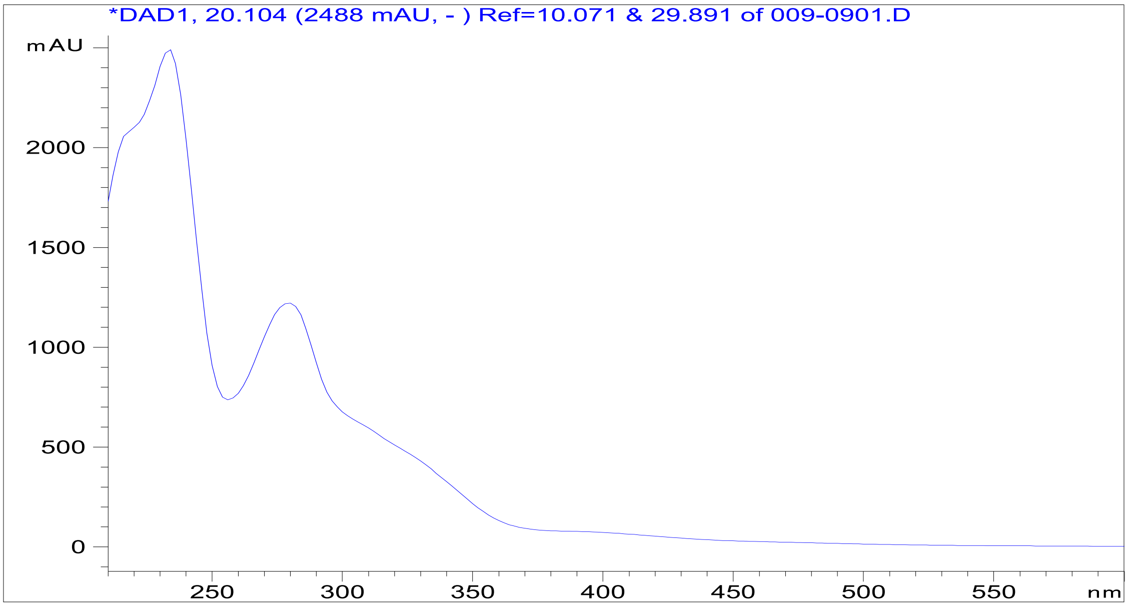

Figure S29. UV-Vis spectrum of compound No 13 (Table 1) eluted at Rt 20.1 min, identified as Daidzein

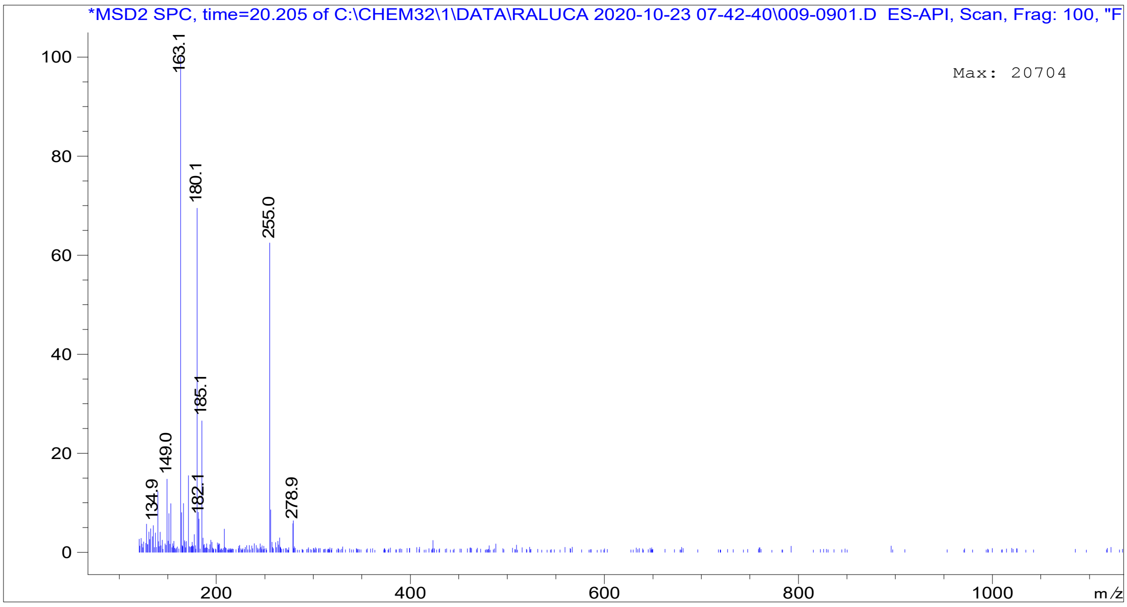

Figure S30. MS spectrum of compound No 13 (Table 1) eluted at Rt 20.1 min, identified as Daidzein

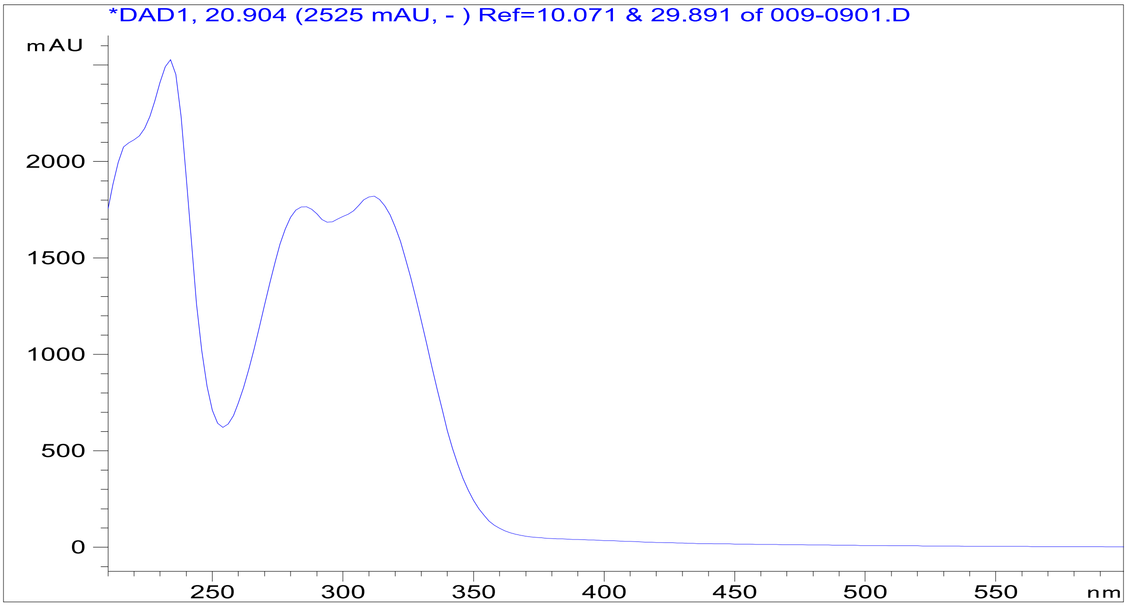

Figure S31. UV-Vis spectrum of compound No 14 (Table 1) eluted at Rt 20.8 min, identified as Catechin rhamnoside

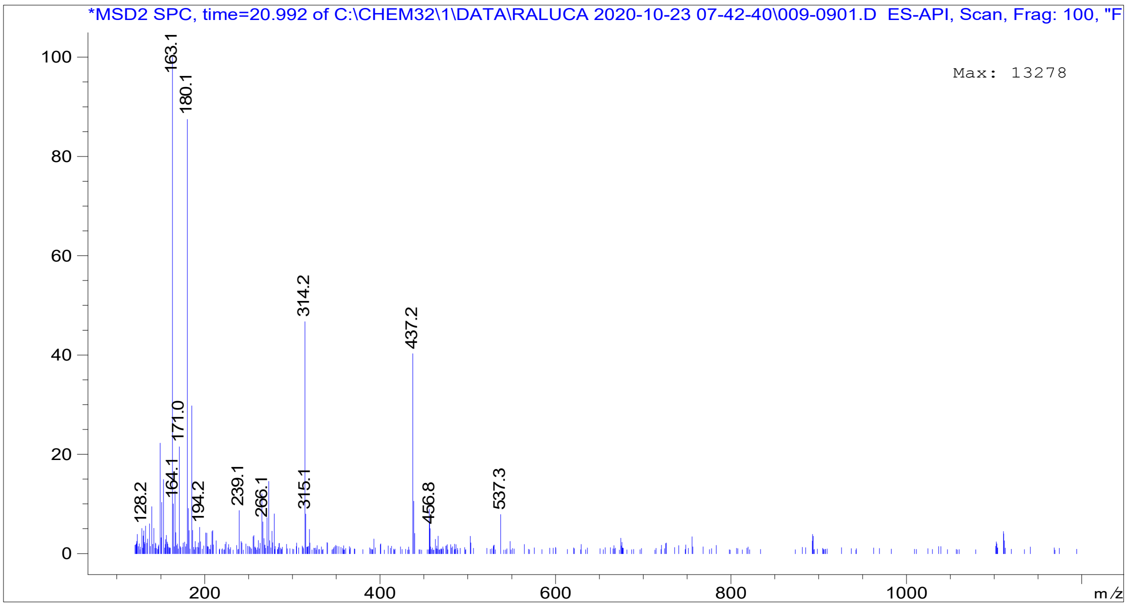

Figure S32. MS spectrum of compound No 14 (Table 1) eluted at Rt 20.8 min, identified as Catechin rhamnoside

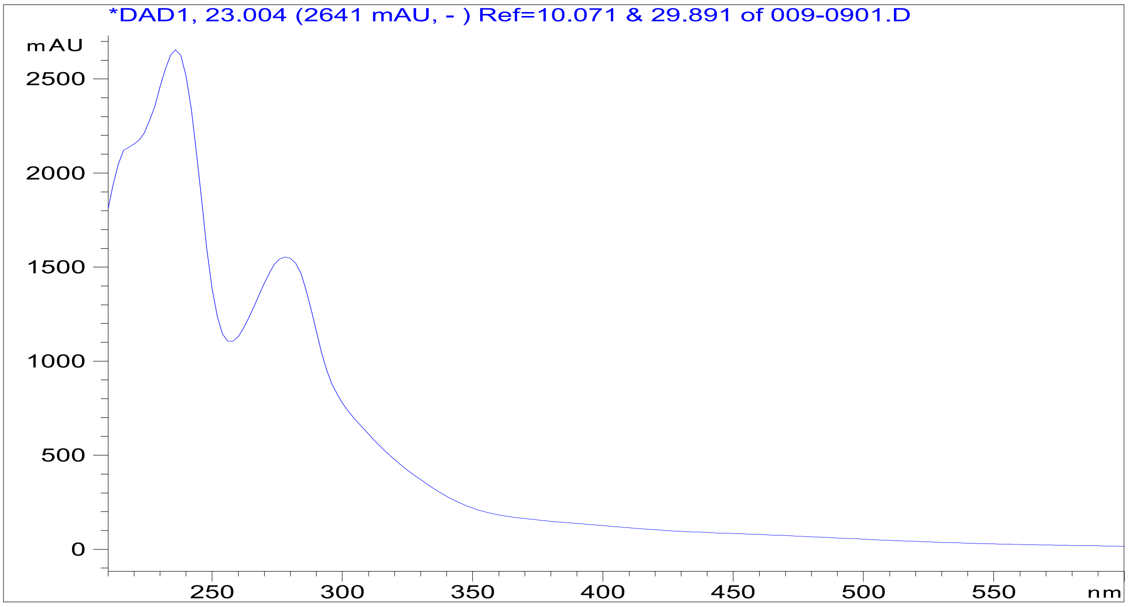

Figure S33. UV-Vis spectrum of compound No 15 (Table 1) eluted at Rt 22.9 min, identified as (Iso)Rhamnetin

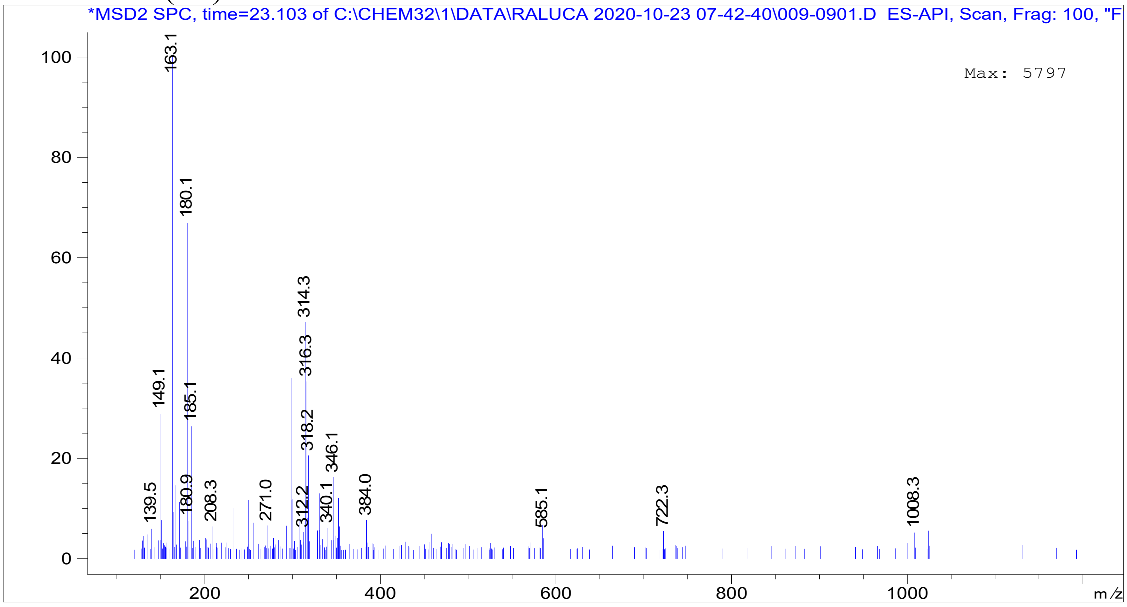

Figure S34. MS spectrum of compound No 15 (Table 1) eluted at Rt 22.9 min, identified as (Iso)Rhamnetin

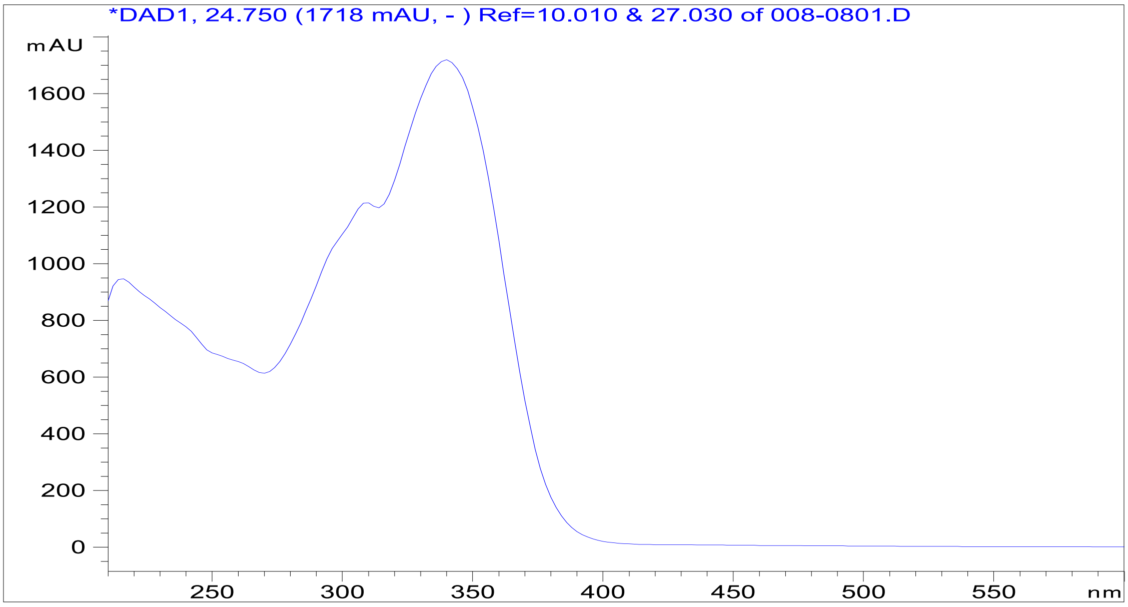

Figure S35. UV-Vis spectrum of compound No 16 (Table 1) eluted at Rt 24.7 min, identified as Kaempferol

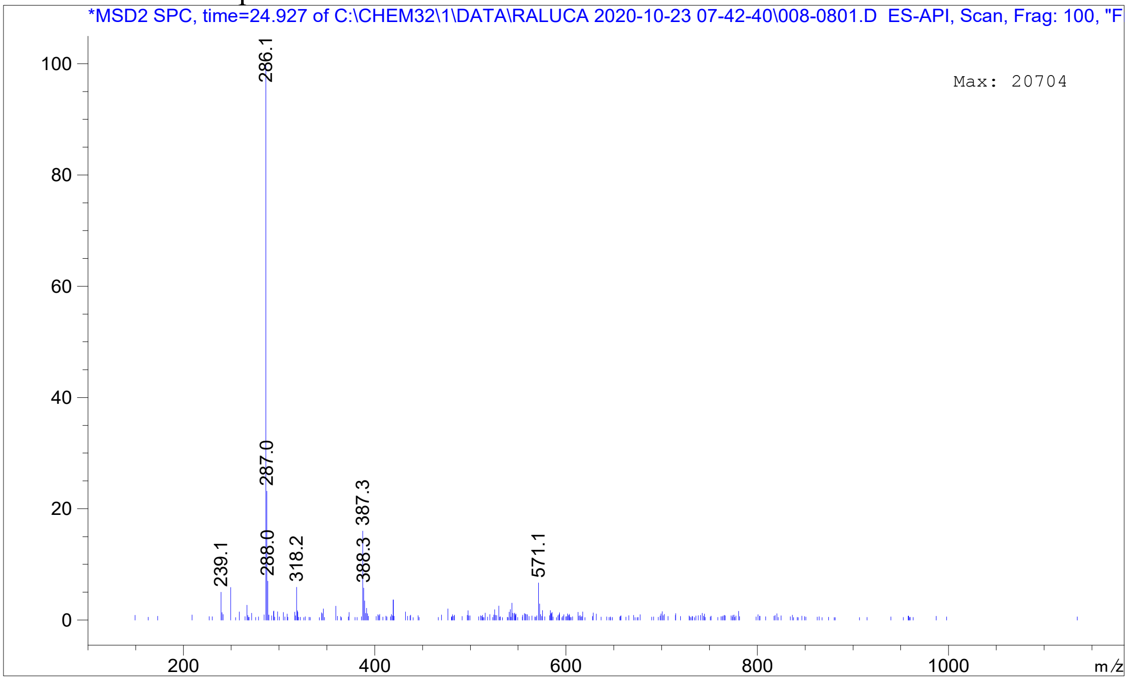

Figure S36. MS spectrum of compound No 16 (Table 1) eluted at Rt 24.7 min, identified as Kaempferol

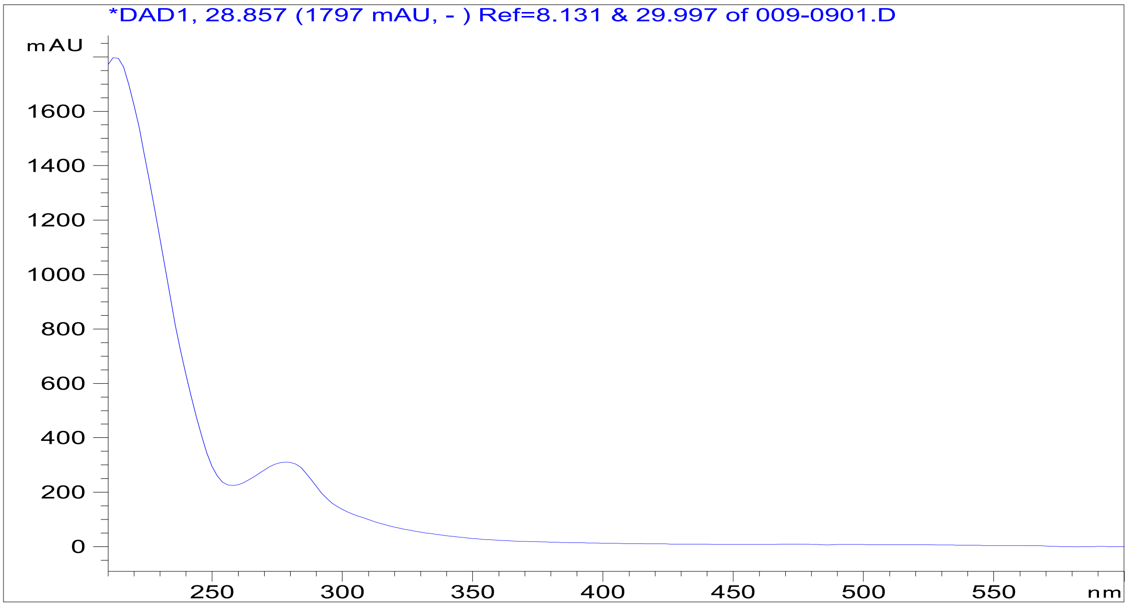

Figure S37. UV-Vis spectrum of compound No 17 (Table 1) eluted at Rt 28.8 min, identified as Proanthocyanidin-tetramer

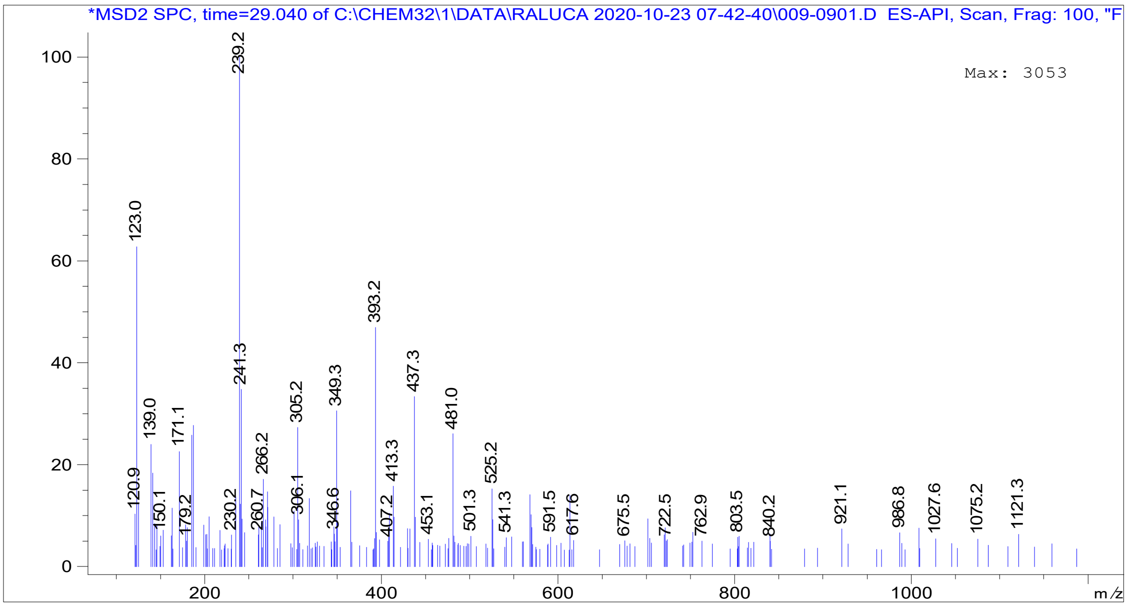

Figure S38. MS spectrum of compound No 17 (Table 1) eluted at Rt 28.8 min, identified as Proanthocyanidin-tetramer
